# The landscape of sex-differential transcriptome and its consequent selection in human adults

**DOI:** 10.1186/s12915-017-0352-z

**Published:** 2017-02-07

**Authors:** Moran Gershoni, Shmuel Pietrokovski

**Affiliations:** Department of Molecular Genetics, Weizmann Institute of Science, Rehovot, Israel

**Keywords:** Sex-differential expression, Sex-differential selection, Sexual dimorphism

## Abstract

**Background:**

The prevalence of several human morbid phenotypes is sometimes much higher than intuitively expected. This can directly arise from the presence of two sexes, male and female, in one species. Men and women have almost identical genomes but are distinctly dimorphic, with dissimilar disease susceptibilities. Sexually dimorphic traits mainly result from differential expression of genes present in both sexes. Such genes can be subject to different, and even opposing, selection constraints in the two sexes. This can impact human evolution by differential selection on mutations with dissimilar effects on the two sexes.

**Results:**

We comprehensively mapped human sex-differential genetic architecture across 53 tissues. Analyzing available RNA-sequencing data from 544 adults revealed thousands of genes differentially expressed in the reproductive tracts and tissues common to both sexes. Sex-differential genes are related to various biological systems, and suggest new insights into the pathophysiology of diverse human diseases. We also identified a significant association between sex-specific gene transcription and reduced selection efficiency and accumulation of deleterious mutations, which might affect the prevalence of different traits and diseases. Interestingly, many of the sex-specific genes that also undergo reduced selection efficiency are essential for successful reproduction in men or women. This seeming paradox might partially explain the high incidence of human infertility.

**Conclusions:**

This work provides a comprehensive overview of the sex-differential transcriptome and its importance to human evolution and human physiology in health and in disease.

**Electronic supplementary material:**

The online version of this article (doi:10.1186/s12915-017-0352-z) contains supplementary material, which is available to authorized users.

## Background

Sexual reproduction is present in nearly all multicellular eukaryotes [1]. In all cases, males and females have identical genetic information across most of their genomes, but harbor many distinct sex-specific characteristics. For example, mammalian offspring depend on maternal lactation in their early life. Lactation is thus a key factor in mammalian reproduction, and its associated genetic system is expected to be under tight selection. However, genes involved in lactation are also carried by males, who do not express this trait [2]. Different selection constraints are thus expected on these genes in males and females. Such cases can lead to reduced purifying selection on genes that otherwise are expected to be highly conserved [3]. In the same manner, many genes that are associated with sexually dimorphic traits might undergo differential selection, which will likely impact reproduction, evolution, and even speciation events [4]. Human sexual dimorphism has been demonstrated for diverse traits, such as brain anatomy and development [5–7], behavior [8], mortality, longevity and morbidity [9, 10], and distribution and metabolism of fat biogenesis [11, 12]. Physical performance capabilities and pain response have also been shown to differ between men and women [13–15]. Previous work found that about 15% of the expression quantitative trait loci (eQTLs) identified in B-lymphocytes have a sex-biased impact on gene expression [16]. That work also reported an overlap of eQTLs and genome-wide association study single nucleotide polymorphisms that are associated with sex-biased diseases. Moreover, a recent work reported sex-specific genetic architecture in complex traits [17]. It is therefore not surprising that men and women differ in their predisposition to many diseases, in disease courses, and in drug response [18, 19]. Manifestations of all these differences are likely associated with the biology of sexual reproduction.

Sexual dimorphism was suggested to evolve due to differential selection on equally expressed traits that become sexually dimorphic and even sex-limited traits [20]. This can lead to the accumulation of genes with different effects on males and females. It is thus expected that the vast majority of sexually dimorphic traits are due to differential expression of genes that are present in both sexes [21]. While carried by both males and females, such genes are expected to undergo sex-biased selection. This can lead to diverse selection patterns, including sexual antagonism where alleles increasing the fitness in one sex reduce it in the other [21]. In population genetics terms, the cost of sexual dimorphisms might be reflected in the elevated frequency of an allele with deleterious effects only on one sex. Hence, a mutation causing congenital disease in only one sex can propagate to a high population frequency due to reduced selective constraints or neutrality in half of the population (i.e*.*, in the other sex). This might contribute to sex specificity in the susceptibility to common diseases, and provide a partial explanation to the phenomenon of “missing heritability” [18]. Indeed, differential selection due to sexual dimorphism was suggested and modeled as a mechanism that contributes to the propagation of deleterious mutations in the population [22, 23]. We recently showed first evidence that this occurs in humans. We found that deleterious mutations in testis-exclusive genes tended to accumulate more than expected, likely due to reduced selective constraints in women [24]. However, a more general demonstration of the association between sex-differential gene expression and sex-differential selection is limited to model organisms [25], mainly due to poor mapping of the sex genetic architecture and the unavailability of large-scale transcriptome sequencing in humans [24, 26].

Mapping sex-differential selection and gene expression are fundamental for understanding human evolution and biology, in health and disease. Recent advances in DNA sequencing technologies with steadily dropping costs have made such aims feasible. The release of the Genotype-Tissue Expression (GTEx) project data, which currently includes 53 tissue samples from 544 donors [27, 28], has paved the way for such progress, and preliminary results for sex-differential gene expression are already available [28].

Here, by rigorous analysis of RNA-sequencing (RNA-seq) data from the GTEx project [27, 28], we have comprehensively mapped, for the first time, human adults sex-differential gene expression over 45 tissues common to both sexes. We then identified highly and moderately sex-specific genes while considering the complete panel of 53 tissues. Such genes are expected to have general sex-differential roles, thus suggesting differential selection. We thus hypothesized that deleterious mutations in these genes will propagate in the population more than expected by chance, due to the reduced impact of purifying selection [22, 24, 29]. By analyzing the signature of selection in these genes, we have found, for the first time, reduced selective constraints and differential rates of accumulation of deleterious mutations in both men and women sex-specific genes. The expression and function of these genes are associated with several tissues and biological pathways, including ones common to both sexes, suggesting a general phenomenon that directly arises from sex-differential selection. Moreover, many of these sex-differentially expressed genes were enriched in sexually dimorphic systems. Finally, some of these genes suggest new insights into the pathophysiology of several human diseases.

## Results

We examined human gene expression from RNA-seq data of the GTEx project version 6 (October 2015 release), including 8555 samples comprising 53 tissues from 357 men and 187 women post-mortem donors aged 20–79 years old [30]. Gene expression data for each tissue was grouped by sex. This created 98 sets with 45 tissues common to men and women and eight tissues specific to one of the sexes.

### Sex-differentially expressed genes

Sex-differential expression (SDE) was tested in each of the 45 common tissues by comparing the individual expression values of 18,670 out of 19,644 informative protein-coding genes in men versus women. To identify SDE we used the NOISeqBIO method [31, 32] to compare gene expression in the common tissues between men and women. The results were further analyzed to produce a relative SDE score for each gene in each common tissue using a metric we devised (Additional file 1: Figure S1).

On the background of similar expression in most tissues of most genes (Additional file 2: Figure S2; Additional file 3: Table S1), there are over 6500 protein-coding genes with significant SDE in at least one tissue. Most of these genes have SDE in just one tissue, but about 650 have SDE in two or more tissues, 31 have SDE in more than five tissues, and 22 have SDE in nine or more tissues (Additional file 4: Figure S3 and Additional file 5: Table S2). As expected, Y-linked genes that are normally carried only by men show SDE in many tissues. Nevertheless, 16 out of the 244 X-linked SDE genes also have widespread SDE (across six or more tissues, Additional file 5: Table S2) in either men or women. We found that three of these X-linked genes are located at pseudo-autosomal region 1 (PAR1), which undergoes relatively frequent recombination between the X and Y chromosomes and is known to escape X-inactivation [33] (Additional file 5: Table S2; Additional file 6: Figure S4). It is noteworthy that these PAR1 genes have men-biased expression.

**Fig. 1:**
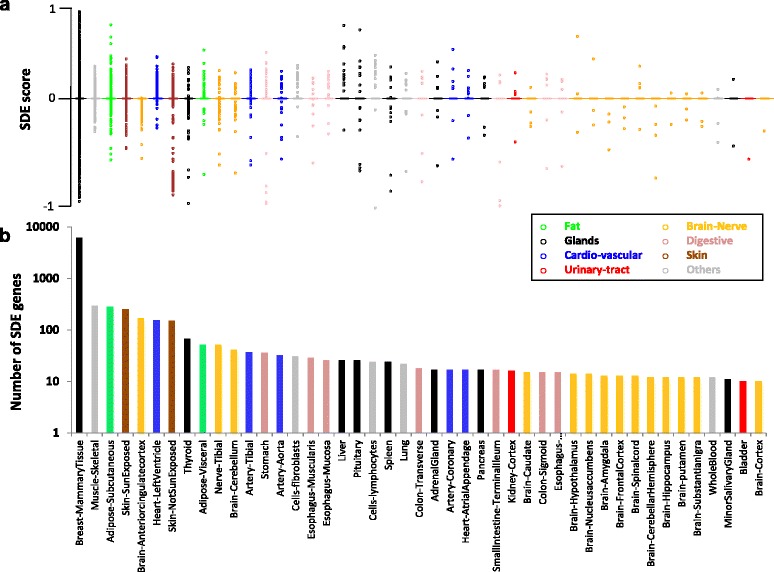
Box plot of (**a**) sex-differential expression (*SDE*) scores of all protein-coding genes, and (**b**) the number of SDE genes in 45 tissues common to men and women. Most genes are not differentially expressed, and have an SDE score of zero. Positive and negative values denote women- and men-biased expression, respectively, colored according to their organs or their biological-system affiliation

**Fig. 2:**
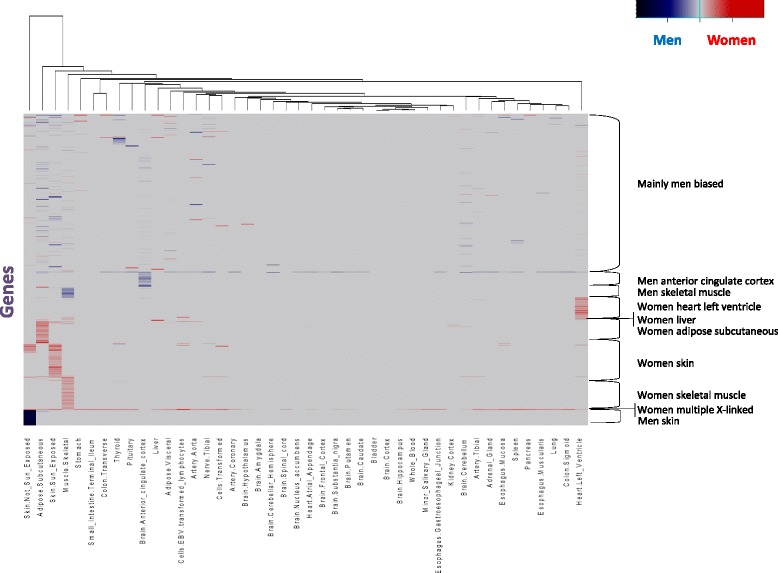
Heatmap of sex-differential expression (SDE) scores of all genes with at least one SDE in non-mammary gland tissue. *Red* denotes women specificity and *blue* denotes men specificity. The genes are grouped according to principal component analysis clusters (Additional file 8: Figure S6). Tissues are grouped using hierarchical clustering

The most sex-differentiated tissue, with 6123 SDE protein-coding genes, is the breast mammary glands (Fig. 1; Additional file 2: Figure S2), as previously noted [28]. This suggests major differences in the physiology and sex genetic architecture of this tissue. We found 1145 genes to be SDE in non-mammary gland tissues. The most differentiated of these tissues, with over 100 SDE genes, are the skeletal muscle, two skin tissues, subcutaneous adipose, anterior cingulate cortex, and heart left ventricle (Figs. 1 and 2). Most GTEx tissues (46 out of 53) have more than seventy samples (70–361). This sample-size variation can affect the number of identified SDE genes per tissue. The Pearson correlation coefficient between the sample size and the number of identified SDE genes is 0.10 for the 45 analyzed tissues common to men and women, and 0.57 when the mammary glands tissue is excluded. This suggests that sample size contributes to the differences in the number of identified SDE genes per tissue, although several tissues noticeably deviate from this trend (e.g*.*, the breast and whole blood tissues, Fig. 1). Besides the number of SDE genes, the tissues can also be clustered by the patterns of gene SDE scores. This analysis found the two skin, adipose-subcutaneous, and stomach tissues to deviate the most from all other tissues, and that seven of the thirteen brain tissues clustered together (Fig. 2, Additional file 7: Figure S5) [34].

Clustering genes by their SDE patterns across tissues revealed 10 groups (Fig. 2, Additional file 8: Figure S6), nine of which can be described as follows:

1. Three groups of men-biased expression in the skin, skeletal muscle, or cingulate cortex tissues (e.g.*, MYH1*; Fig. 3).

**Fig. 3:**
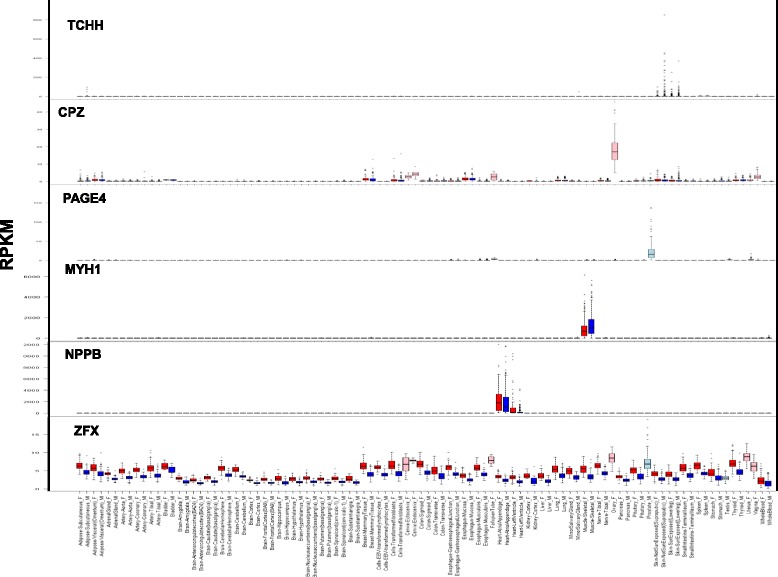
Examples of various patterns of differential expression. Expression of genes *TCHH*, *CPZ*, *PAGE4*, *MYH1*, *NPPB*, and *ZFX* in 53 human tissues. Reads per kilobase of transcript per million values of these genes were retrieved from the GTEx project data [27, 28]. *Red bars* denote women samples and *blue bars* denote men samples; *pink bars* denote women reproductive tissues and *light blue bars* denote men reproductive tissues
2. Five groups of women-biased expression in the liver, heart left ventricle, skin, skeletal muscle, or adipose subcutaneous tissues (e.g., *NPPB*; Fig. 3).
3. A group of mostly X-linked genes with SDE in various tissues, mainly with women-biased expression (e.g., *ZFX*; Fig. 3).

Other genes, such as *TSHB*, show tissue-specific expression bias (Additional file 9: Figure S7), and a few genes present an alternating pattern of expression biases, such as *MUCL1* that is overexpressed in men skin tissue and in women mammary glands (Additional file 9: Figure S7). To detect differential expression in genes with complex modes of expression we used an additional analysis approach, which is more sensitive to such cases. This analysis uncovered 241 additional genes in non-mammary gland tissues that were clearly not detected in the first approach (see “Methods” and Additional file 10: Table S3, supplementary results). For instance, we found a likely age-related gene overexpression in women brain tissue (Additional files 11 and 12: Figures S8 and S9).

Genes found to have SDE were analyzed for gene enrichment in different types of terms (e.g., diseases, Gene Ontology (GO) terms, pathways [35]). Genes with women-biased expression were associated with obesity, muscular diseases, and cardiomyopathy. In addition, overexpressed women-biased genes were enriched in glucose metabolism and adipogenesis pathways (Additional file 13: Table S4). Interestingly, 15 out of 20 genes found to be associated with cardiomyopathy also showed a women overexpression bias in heart tissue, as in the natriuretic peptide B-secreted cardiac hormone gene *NPPB* (Fig. 3), supporting previous evidence on its involvement in sex-differential cardiovascular phenotypes [36, 37]. Genes with men-biased expression also showed enrichment in glucose metabolism pathways, but the gene sets differed, suggesting alternative pathways in glucose metabolism between men and women (Additional file 14: Table S5). A muscle-contraction pathway was also associated with genes overexpressed in men (Additional file 14: Table S5). This might be related to the physiological differences in muscle tissues and in physical features between men and women [38, 39].

### Identification of sex-specific genes

**Fig. 4:**
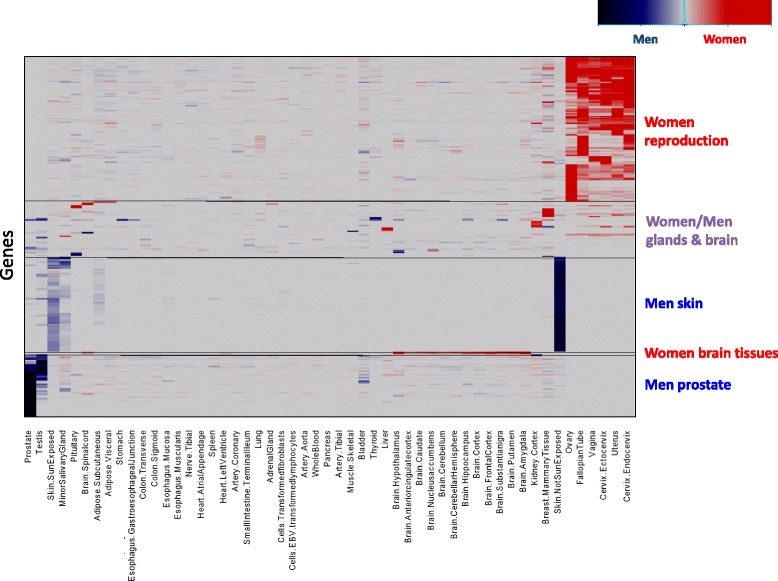
Heatmap of sex-differential expression (*SDE*) scores of the sex-specific and moderately sex-specific genes, colored as in Fig. 2. *Red*, *blue*, and *purple boxes* denote major women, men, and combined gene clusters, respectively

Beyond genes that have SDE in one or several tissues are more extreme cases of genes with overall exclusive or high expression-specificity in one sex [40]. Such sex-specific genes are more likely to have global sex-differential functional roles, and are thus expected to present measurable sex-differential selection that can be reflected by a reduction in purifying selection [24]. A gene was considered sex specific if its maximal expression value in one sex was significantly higher from its expression values in all tissues of the other sex. In addition, genes were considered as non-SDE if their maximal expression values in men and women differed by no more than 10% (≤1.1 fold). We identified 1559 sex-specific and moderately sex-specific genes. Of these genes, 1288 (82.6%) were men-specific and overexpressed in the testis (Additional file 15: Table S6; Additional file 16: Figure S10). Aside from these 1559 genes, we found 26 women-specific and 114 moderately women-specific genes, and 82 non-testis men-specific and 49 moderately men-specific genes (Fig. 4; Additional file 17: Table S7). Over 8000 genes were identified as non-SDE (see “Methods” and Additional file 3: Table S1).

The sex-specific and moderately sex-specific genes could be grouped by their expression patterns into six major categories (Fig. 4; Additional file 16: Figure S10):

1. Testis overexpressed genes in men (Additional file 16: Figure S10)
2. Prostate overexpressed genes in men (e.g., *PAGE4*, Fig. 3)
3. Reproductive system overexpressed genes in women (e.g., *CPZ*, Fig. 3)
4. Skin-specific overexpressed genes in men (e.g., *TCHH*, Fig. 3)
5. Brain tissue overexpressed genes in women
6. Mainly gland and brain tissue overexpressed genes, in men or women (e.g., *TSHB*, Additional file 17: Table S7).

Overall, sex-specific genes are mainly expressed in the reproductive system, emphasizing the notable physiological distinction between men and women. However, scores of genes that are not known to directly associate with reproduction were also found to have sex-specific expression (e.g., the men-specific skin genes).

### Selection analysis

We calculated the numbers of observed (1000 Genomes Project [41]) and possible deleterious non-synonymous (DNS), stop-gain, and synonymous (S) single-nucleotide variants (SNVs) for each gene. This allowed us to quantify the selection pressure and its direction by dDNS/dS and dStop/dS ratios. Similar to dN/dS, these ratios are selection indicators [42, 43]. Ratios close to 1 indicate neutral selection, lower ratios indicate purifying (negative) selection, and significantly higher ratios suggest adaptive (positive) selection (see “Methods”).

**Fig. 5:**
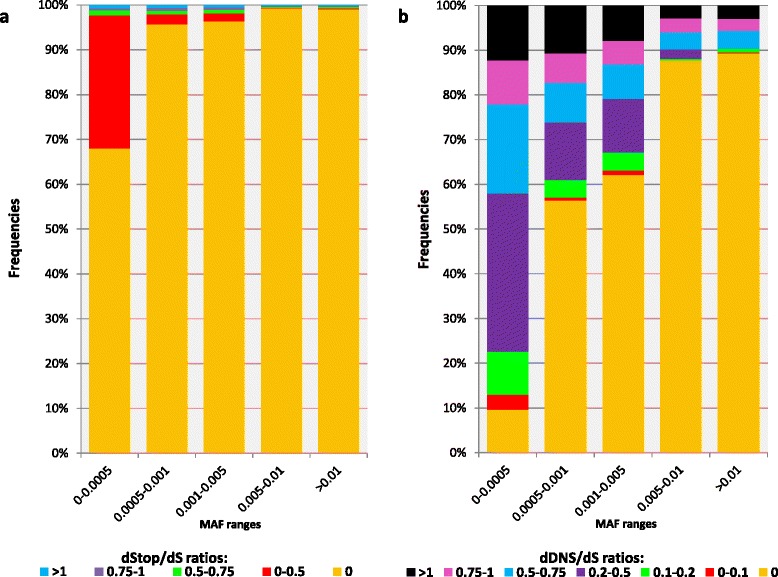
Population genetics of selection pressures. Population distribution frequencies (y-axis) of protein-coding gene (**a**) dStop/dS and (**b**) dDNS/dS values in the 1000 Genome Project, Phase 3, for different minor allele frequency (*MAF*) ranges (x-axis). Different dStop/dS and dDNS/dS ratio ranges are denoted by different colors (see key). *dDNS* deleterious non-synonymous, *dS* deleterious synonymous, *dSTOP* deleterious stop-gain

Natural gene variants have different frequencies, with most of the variation due to alleles with rare to low minor allele frequencies (MAFs) [24, 44]. However, selection is expected to have only a slight effect on the propagation of very rare variations because they are predominantly new while selection is mainly a long-term process [44, 45]. In addition, most phenotypes result from allele and gene interplay, and are thus highly unlikely (except in inbreeding) for rare variations, as in recessive and epistatic models of inheritance [45]. We hence studied the population genetics of the dDNS/dS and dStop/dS to find the proper MAF threshold in which the selection efficiency is maximal. Higher dDNS/dS ratios are more abundant for SNVs with rare MAFs (<0.005, Fig. 5), indicating that negative selection predominantly affects the propagation of deleterious SNVs for MAFs >0.005, as previously shown [24, 44]. However, dStop/dS ratios are sharply decreased for very rare SNVs (i.e*.*, MAFs <0.001, Fig. 5). We thus further analyzed the effect of selection on DNS and stop-gain using MAF thresholds of >0.005 and >0.001, respectively.

### Selection analyses of sex-specific and moderately sex-specific genes

**Fig. 6:**
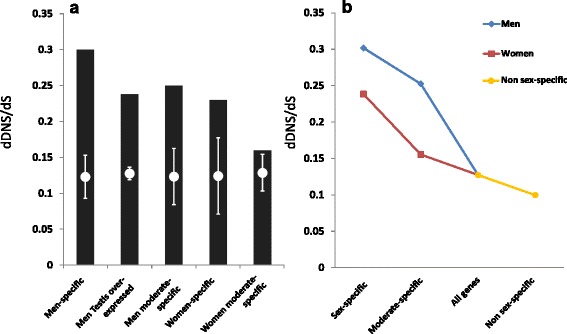
Sex-specific expression and purifying selection. **a** dDNS/dS ratios of different groups of genes (Table 1, *black bars*) and the mean (*white circles*) and standard deviations (*lines*) of 10,000 random control sets with the corresponding number of genes. **b** Inverse correlation between sex specificity and selection efficiency. *dDNS* deleterious non-synonymous, *dS* deleterious synonymous

We have previously shown that human testis-exclusive genes are under reduced selection [24]. All 1100 of 1295 men testis-overexpressed genes identified here that are covered in the 1000 Genomes Project were also found to have significantly higher dDNS/dS and dStop/dS ratios (Table 1). This gene set includes 77 out of 95 of the genes we previously identified as testis exclusive [24]. The other 18 out of 95 genes that we previously found to be specifically expressed in testis tissues might not be identified here because these tissues are not present in the GTEx samples. The non-testis men-specific and moderately women-specific genes also had significantly higher dDNS/dS ratios (Table 1, Fig. 6). The significantly higher dDNS/dS ratio of these men-specific genes did not depend on the presence of the 55 keratin genes (Table 1). women-specific genes too had a significantly higher dDNS/dS ratio (Table 1, Fig. 6). Moderately women-specific genes had a higher, yet not significant, dDNS/dS ratio (Table 1). However, when comparing the moderately women-specific genes to non sex-specific genes, we found the dDNS ratios to be significantly higher for the moderately women-specific genes (1.66 fold change, Fisher’s exact test *p*-value <1 × 10^−4^) but the dS ratios showed no significant change (1.08 fold change, Fisher’s exact test *p*-value = 1.5 × 10^−1^). Thus, moderately women-specific genes have significantly reduced selection relative to non sex-specific genes. The same analysis for dStop/dS of men- and women-specific genes also found significantly reduced selection (Table 1). A significant reduction in purifying selection on sex-specific genes was hence found by independent analyses of selection on DNS and stop-gain mutations on diverse sets of sex-specific genes from both women and men, including sets from non-reproduction-related tissues. It is also notable that although reduced selection was observed for both men- and women-specific genes, it was higher in men-specific genes compared to women-specific genes (Fig. 6, Table 1).

**Table 1:** Selection analysis summary

Gene group	*n*	dDNS/dS (MAF > 0.005)	*p*-value	dStop/dS (MAF > 0.001)	*p*-value
Women-specific	26	0.23	0.02	0.27	0.0117
Men-specific	82	0.30	0.0005	0.29	0.0001
Men-specific; no keratin and keratin-associated genes	27	0.28	0.009	0.22	0.026
Moderately women-specific	114	0.16	0.09	0.13	0.0076
Moderately men-specific	49	0.25	0.005	0.07	0.27
Men testis overexpressed	1100	0.238	<0.0001	0.29	<0.0001

*dDNS* deleterious non-synonymous, *dS* deleterious synonymous, *dSTOP* deleterious stop-gain, *MAF* minor allele frequency

## Discussion

Mapping sex-differential gene expression we found more than 6500 protein-coding genes with significant SDE in one tissue or more. The most differentiated tissue was the breast mammary gland, with more than 6000 genes having significant SDE (Fig. 1). This remarkable sex-biased gene expression is likely due to the distinct physiologic properties of this tissue between men and women [2]. In evolutionary terms, differential selection between the sexes of so many genes that are likely involved in lactation, an essential reproductive trait, might inhibit optimal adaptation of this trait due to its distinct importance in men and women.

Almost all SDE genes are sex differentiated in one or just a few tissues. Thirty-one genes have SDE in six or more tissues. Besides Y-linked genes that have men-specific expression, 16 of the other genes are X-linked, with multiple-tissue SDE in either men or women. Three of these X-linked genes are located in the PAR1 region (Additional file 6: Figure S4; Additional file 5: Table S2), which includes genes that undergo recombination with the Y chromosome and also escape X-inactivation [33]. These PAR1 genes have identical sequences in their X and Y copies (Additional file 5: Table S2), but are only classified as X-linked in the GTEx data. While this should have led to similar expression in men and women (as in most autosomal genes), these genes have men-biased expression in multiple tissues. It is possible that although the copies are identical, the regulation of their expression is distinct between the X and Y-chromosomes. Besides the PAR1 genes, X-linked SDE genes in multiple tissues were found to only have women-biased expression (Additional file 6: Figure S4). In several cases we found that such genes have an active paralog on the Y chromosome and it is therefore likely that these genes escape X-inactivation and both X alleles are expressed in women, while men have only one X-linked allele.

Aside from the mammary glands, the adipose, skeletal muscle, skin, and heart tissues have over a one hundred SDE genes. This indicates substantial differences in the physiology, or alternate biological pathways, in these tissues between adult men and women. However, the differences in the number of SDE genes per tissue should be carefully assessed because the variability in tissue sample sizes could contribute to the number of SDE genes per tissue that we can identify. Functional terms analysis of SDE genes suggests sexual dimorphism in fat biogenesis, muscle contraction, and cardiomyopathy (Additional files 13 and 14: Tables S4 and S5). Tissues with few identified SDE genes might have overall similar function between men and women, yet even very few SDE genes can have extensive physiological impacts on the organism. For instance, the pituitary gland has only 26 identified SDE genes (Figs. 1 and 2), but two of them are the FSHB (women-biased) and TSHB (men-biased) gonadotropin hormones that have wide-ranging roles in human reproduction and metabolism [46, 47]. Another example is the CYP3A4 and CYP2B6 cytochrome P450 enzymes, which have women-biased expression in liver. Cytochrome P450 (P450, CYP) enzymes are associated with drug metabolism and other essential catabolic processes [48], and might be involved in sex-differential drug responses, as previously reported [49]. Other identified specific genes might shed new light on the pathophysiology of human diseases. For instance, the *NPPB* gene, which is mainly overexpressed in young women’s hearts (Additional file 18: Figure S13), is related to cardiovascular homeostasis [36, 37]. Variations in this gene are associated with postmenopausal osteoporosis, a health condition mainly affecting women [50]. Thus, a sexually dimorphic effect of this gene on both phenotypes would be interesting to assess.

To evaluate the association between SDE and selection we identified sex-specific genes. Such genes are likely to possess different roles between the sexes and therefore are likely to undergo different selection pressures in each sex. The vast majority of sex-specific genes we found are overexpressed in the testis. We previously showed reduced selection and accumulation of damaging mutation in such genes. Here we confirmed our previous findings, extended them to many more testis-overexpressed genes, and to sex-specific genes of other men and women tissues. Many of the non-testis sex-specific genes are also related to the reproductive system, including genes expressed in tissues common to both sexes, such as gonadotropin hormones expressed in the pituitary (e.g., *FSHB* and *CGB7*). Dozens of genes with no direct association to reproduction were also identified as sex specific. Many of these genes are expressed in skin tissues, are linked to hairiness (Additional files 13 and 14: Tables S4 and S5), and are likely involved in hair dimorphism in women and men. Other non-reproductive genes do not seem to share common features with each other, but are each interesting on their own, for example, the moderately men-specific growth hormone GHRH and the men-specific calcitonin-related polypeptide alpha (CALCA) (Additional file 17: Table S7). The latter is involved in calcium regulation and functions as a vasodilator [51, 52]. The genes fro both seem specific to adult men, although they are related to apparently general biological processes.

Analyzing selection on highly and moderately men- and women-specific genes, we found a significant association with reduced selection efficiency, as reflected in their dDNS/dS and dStop/dS ratios (Table 1, Fig. 6). The reduced purifying selection efficiency was also correlated with the level of sex specificity. This suggests that higher sex specificity indicates greater distinction in the functional importance for each sex, and reduced selection efficiency. This in turn enables the propagation of damaging alleles through the non-expressing sex lineages. The resulting relatively high population frequencies of these alleles can enhance the prevalence of different human diseases.

Although we found reduced selection on both men- and women-specific genes, it is notable that reduced selection was more prevalent in men-specific genes (Fig. 6). This supports our previous expectations to find men-specific genes to be under less selection than women-specific genes [24]. We suggest that the basis for this could be the practically unlimited numbers of available male gametes compared to the restricted number of available women gametes, as suggested in the Bateman principle [53]. Thus, the ability of women to pass on alleles that cause men-specific lethality will less affect the number of fertile men required to sustain the population, but not vice versa.

In this work we focused on protein-coding genes, because currently there is a broad functional knowledge on these genes and extensive experience in analyzing and quantifying the selection trends these genes have undergone. However, the importance of non-coding RNA genes for the regulation and execution of sexual dimorphism was not ignored. For instance, the function of the *XIST* long non-coding RNA gene in the sex-specific X-inactivation process is well documented (Additional file 19: Figure S11) [54]. Our preliminary observations of the RNA gene differential transcriptome support a global role of these genes in the sex genetic architecture (Additional file 20: Figure S12). Hence, this work and the data it provides might trigger further in-depth studies on the contribution of RNA genes to sexual dimorphism.

Finally, the vast majority of sex-specific genes we found are associated with the reproductive system. Damaging mutations in many reproductive genes can hence propagate to high population frequencies. We suggest that sex-specific genes are major contributors to the high incidence of infertility in men and women.

Our results are delimited by the scope of the data in the GTEx study. This study includes 53 tissues from adult humans. All tissues are composed of several cell types and a few are represented in fewer than 15 men or women donors. We believe our statistical and analysis measures excluded most false-positive results. However, the distinct age limits of the samples are acutely pertinent to sexual dimorphism and we do not know how much of our findings can be extended beyond adults. Examining comparable data from puberty and during embryonic stages of sex determination will likely augment the genes and phenomena described here.

After submitting this work for review, two studies on sexual dimorphism in human gene expression were made public. Kassam *et al*. examined the sex-specific genetic architecture of autosomal gene expression in whole blood samples from about one thousand men and one thousand women using DNA arrays [55]. No differences between men and women were found in autosomal genetic control of gene expression. We too did not identify autosomal genes with different expression between men and women in the GTEx whole blood tissue (Fig. 1; Additional file 3: Table S1). Chen *et al.* posted to bioRxiv a non-peer-reviewed preprint analyzing the GTEx data for gene expression sexual dimorphism and regulatory networks [56]. They report sexually dimorphic patterns of gene expression involving as many as 60% of autosomal genes. Similar to our findings, they reported breast, skin, adipose, heart, and skeletal muscle as the most sexually dimorphic tissues. The studies vary in their analyses procedures and emphasize different contexts of SDE. These studies are complementary works with different insights.

The mode of gene expression is very complex, depending on the gene’s genomic and chromatin contexts, activity of other genes, expressing tissue, the individual’s developmental stage, and external factors such as exposure to pathogens, diet, and temperature. The expression level of genes thus varies temporally (in scales of minutes to decades) and across tissues, and is a multidimensional system. This is the key challenge in evaluating differential gene expression between populations.

SDE between men and women stems from any deviations of gene activity in place (i.e., organs, tissues, and cells) and time (e.g., developmental stage, age, cell cycle point, or periodic processes). The overall distribution of gene expression values in two populations could be highly similar, and distinct in only a minor subset of samples that represents a genuine biological difference in time and/or place. For instance, a gene can have similar basal expression in men and women, but upon sex-specific induction its expression will be altered only in one sex. Thus, only a small fraction of one population in any one time might differentially express this gene. Identifying differential expression is thus a challenging problem. In addition, sex-specific expression is a particular case of SDE, in which genes present a global bias in their mode of expression in one sex compared to the other.

We applied several approaches to identify SDE and sex-specific expression. Besides analyzing differences according to the population variance (NOISeqBIO), we also used an approach that gave weight to a subset of samples that notably deviated from all other samples (using count trimmed means and NOISeq-sim). The DESeq2 method was also used to validate the results in selected datasets. In addition we used a new normalized measure for gene differential expression between pairs of sample populations. This differential expression measure takes into account the expression difference between the sexes and the maximal expression of the gene in all tissues, placing the difference in specific tissues in the context of the gene overall mode of expression. This measure is general and can be used in other population-based differential gene expression studies (Additional file 1: Figure S1). Combining these approaches increased our ability to identify differential expression from various modes of gene expression. Accumulation of many more samples from different donors and conditions will uncover the full spectra of gene modes of expression and improve the resolution of differential expression analyses.

## Conclusions

This work comprehensively mapped for the first time the sex-specific genetic architecture of human adults. We identified hundreds of genes with women and men SDE, and showed the relation of these genes to several sexually dimorphic features, to human diseases, and to human evolution. Our results can facilitate the understanding of diverse biological characteristics in the context of sex. We also demonstrated the increased propagation of deleterious mutations in many men- and women-specific genes and thus the likely contribution of SDE genes to the occurrence of common human diseases.

## Methods

### Data sources

RNA-seq data were retrieved from the GTEx project version 6 [27, 28]. Population variation data were retrieved from the 1000 Genomes Project, Phase 3 (*n* = 2504 individuals, [41]). The human GRCh37 release coding exome coordinates and sequences were retrieved from Ensembl [57].

### Variation analysis

The AnnoVar software package [58] was used to annotate the reported variations from the 1000 Genomes Project, and all possible variations (relative to the GRCh37; h19 reference genome) in every human protein-coding position documented in GRCh37. For each variation we determined its specific protein-transcript consequences, its population frequency, and its predicted functional likelihood (using both SIFT and PolyPhen algorithms [59, 60]). A non-synonymous (NS) variation was considered functional only when both SIFT and PolyPhen algorithms predicted it as deleterious [24]. Because SIFT mainly uses sequence conservation and PolyPhen mainly uses structural and functional impacts, we found the combination of the two methods to be highly accurate (number of true positive from total positive prediction [24]). This analysis calculated the distribution of all mutation types for each gene as observed in the 1000 Genomes Project population, and the computed distribution of all possible nucleotide substitutions consequences (i.e*.*, NS, DNS, S, and stop-gain) for each protein-coding gene. The obtained data allowed us to calculate the deleterious (dDNS), loss-of-function (dStop-gain), and neutral (dS) mutation rates for each gene or group of genes according to the 1000 Genomes Project data. We examined the use of other available sources of human genetic variations, such as ExAC [61]. However, the number of additional SNVs with population frequencies >0.005, which are predominantly affected by selection, from these sources was negligible relative to the 1000 Genomes Project data (not shown).

### Selection analysis

Previously, others and we have shown that the effect of selection on a mutation largely depends on its population frequency. Selection predominantly affects mutations that are have a population frequency >0.005, while very rare mutations (population frequency <0.001) tend to undergo negligible selection [24, 44]. Selection was thus analyzed according to the MAF range of the variations [24]. Selection pressures were assessed by calculating for each gene, or group of genes, the ratios of its functional (DNS and stop-gain) mutation rates to its neutral (S) mutation rate. The rate of a mutation type is the number of observed mutations from a certain type (e.g., S) in the 1000 Genomes Project, Phase 3, divided by all computed possible nucleotide substitutions leading to that type of mutation in the gene. The selection signature is the ratio of the functional rates (dDNS or dStop-gain) divided by the neutral rate (dS), that is, dDNS/dS and dStop-gain/dS. These measures extend the dN/dS type measures, similar to a previous work [43]. As in dN/dS, higher ratios indicate lower purifying selection [42]. To calculate if the dDNS/dS and dStop/dS ratios in a group of sex-specific genes deviated from these ratios in other protein-coding genes, we performed a randomization test: all non-Y-linked, non-testis-specific unique protein-coding human genes for which we have variation data in the 1000 Genomes Project and expression data in the GTEx project were used to create 10,000 random sets for each gene group. The number of genes in each set was the number of genes in the examined gene group. To compare the dDNS and the dS ratios between the two independent groups of moderately women-specific genes and non-sex-specific genes, we performed a Fisher’s exact test.

### Differential expression

Genes with SDE were detected by two approaches from the NOISeq R package [31, 32] The first approach used the NOISeqBIO algorithm, which treats the sample population as biological replicates in which the computed variability within the population is considered as noise [31, 32]. We used this to compare gene reads per kilobase of transcript per million mapped reads (RPKM) expression values between women and men population samples from corresponding tissues after excluding uninformative genes, that is, genes that did not have at least an expression of 1 RPKM in any sample. A probability cutoff of 0.95 was used to identify genes with significant differential expression, as this cutoff value is considered correct for multiple testing [31, 62]. The NOISeqBIO method provides effective statistics for determining differential expression between two populations. However, this approach regards the population variability as noise and could exclude some genuinely sex-differentiated genes that have complex modes of expression. For instance, genes activated during ovulation are expected to be expressed only in a few women (mainly in women <50 years old and on a few days each month [63]), while not being expressed in most women and in all men samples. The differential expression of such genes will be difficult to identify using a straightforward population analysis. To detect at least some of these cases, we used an additional analysis approach that could identify the difference in such cases.

To overcome this issue, at least partially, a single trimmed mean of all RPKM or read count expression values was calculated for every gene from each tissue sample and sex (men or women) by removing the two most extreme sample values. This removed samples that could have skewed the mean. Assuming the trimmed means of read counts reflect the population expression of a gene in men or women samples, we then computed their differential expression using the NOISeq-sim algorithm [32]. NOISeq-sim relies on the assumption that read counts follow a multinomial distribution, in which the probability for each feature in the multinomial distribution is the probability of a read to map to that feature. This identified an additional list of genes with differential expression that were not identified by NOISeqBIO but had NOIseq-sim probability scores of at least 0.8 and a NOISeqBIO probability score at least 0.2 smaller.

Finally, to assess the reproducibility of SDE analysis by NOISeqBIO we implemented and used another differential expression method, DESeq2 [64], and analyzed the adipose-subcutaneous and liver datasets. We found that after *p-*value adjustment for multiple-testing correction, >92% of the adipose-subcutaneous and liver genes identified as SDE in NOISeqBIO were also found to be SDE by DESeq2.

The possible impact of the sample size on the number of identified SDE genes per tissue was tested by the Pearson correlation co-efficient (r). To assess a possible bias in the age distribution between men and women samples we used the two-sample Kolmogorov–Smirnov test. We found no significant differences in age distribution between men and women.

### Gene and tissue clustering

Patterns of differential expression were analyzed using the following gene differential expression score, calculated for tissues with data for both men and women:

$$ \mathrm{S}\mathrm{D}\mathrm{E}= L O{G}_2\left\{\left(1+ EXP{R_{\boldsymbol{\mathsf{g}},\mathbf{t}}}^{\mathbf{w}}/ MA{X}_{\text{\textit{\textsf{g}}}}\right)/\left(1+ EXP{R_{\boldsymbol{\mathsf{g}},\mathbf{t}}}^{\mathbf{m}}/ MA{X}_{\text{\textit{\textsf{g}}}}\right)\right\} $$

Where *g* is a specific gene, *t* is a specific tissue, and *m* and *w* represent men and women, respectively. EXPR_***g,t***_^***w***^ is the NOIseqBIO-calculated mean RPKM expression value of gene *g* in tissue *t* for women (or for men with the m superscript), and MAX_*g*_ is the maximal NOIseqBIO calculated mean RPKM expression value of gene g in all tissues (including tissues specific for men or women). This score returns the differential expression value of a gene in a specific tissue, relative to the maximal expression of the gene. The value ranges from 1 (exclusive expression in women) to -1 (exclusive expression in men). This formula gives lower scores when expression in the examined gene and tissue are lower than those of the gene in some other tissue and can be generalized to compare the difference between two populations normalizing by a maximal value (Additional file 1: Figure S1).

Hierarchical cluster analyses and principle component analysis (PCA) were performed on a matrix of sex-differential expression (SDE) scores, with values of 0 given to genes that were not found significant in the NOISeq statistical analyses described above. Heatmap and hierarchical cluster analyses used the hclust method of the heatmap.2 R package and the pvclust method [34]. The PCA and the partitioning around medoids analyses used the CLARA and PAM methods of the R cluster package [65], with Euclidean distance measurements. This analysis allowed us to group genes according to their SDE patterns similarity.

### Sex-specific expression

To find genes that are specific or highly specific to one sex, for each non Y-chromosome gene we calculated the ratio of its maximal trimmed mean expression values in one sex to its maximal trimmed mean expression in the other sex. Genes were considered as specific or highly sex-specific for ratios of at least 4-fold, when the lower maximal expression value was at least 1 RPKM. A ratio cutoff of 2-fold was used when the higher maximal expression was at least 1 RPKM but the other lower maximal expression value was very low (<1 RPKM). Other genes with sex ratios of 2–4-fold were considered as having moderately sex-specific expression, and genes with ratios of 1.1–0.9-fold were considered as having sex-similar expression. The statistical significance of the highly sex-specific gene expression was tested using the NOISeqBIO method, comparing samples from the tissue with the highest expression in one sex to samples from the tissue with the highest expression in the other sex.

### Gene enrichment analysis

Gene enrichment analysis was performed using the GeneAnalytics server, which can identify gene enrichment for several terms and data sources, including diseases, pathways, GO terms, and tissue expression [35].

## Additional files

Additional file 1: Figure S1. Differential expression score. Landscape of scores for all possible ratios of men and women expression values using a base 2 logarithm. The formula we derived for SDE score can be generalized to compare the difference between two populations (*x* and *y*) in a certain tissue or condition (*t*), normalizing by a gene (*g*) maximal expression value (MAX*g*). This differential expression score (DES) is a logarithm of the normalized ratios, giving scores between −1 and 1. We use a logarithm base of 2, but other bases (*n*) are possible. The general expression is thus DES = LOG*n*{(1 + (EXPR_*g,*__*t*_^*x*^/MAX*g*) * (*n* − 1))/(1 + (EXPR_*g,*__*t*_^*y*^/MAX*g*) * (*n* − 1))} where EXPR_*g,*__*t*_^*x*^ is the expression value of gene *g* in tissue/condition *t* for population *x*. This score returns the differential expression value of a gene in specific tissue/condition, relative to the maximal expression of the gene. The value ranges from 1 (exclusive expression in *x*) to −1 (exclusive expression in *y*). Larger logarithm bases (*n*) exponentially increase the transitions between exclusive expression (1 and −1) and non-differential (0) scores. (PDF 276 kb)
Additional file 2: Figure S2. SDE score heatmap of all protein-coding genes in 45 tissues common to both sexes. Scores are color-coded from blue (strictly men) to red (strictly women), with non-differential expression in white. Most genes are similarly expressed in most tissues with the exception of the breast mammary gland (more than 6000 SDE genes). (PDF 187 kb)
Additional file 3: Table S1. SDE scores for all protein-coding genes in 45 tissues common to men and women. Genes were analyzed by NOISeqBIO with scores of zero given for genes with insignificant differential expression. Other genes have SDE scores below zero for men-biased expression and above zero for women-biased expression. (CSV 2205 kb)
Additional file 4: Figure S3. Occurrence of genes according to number and exact (**a**) or cumulative (**b**) number of tissues they have SDE in. Most SDE genes are differentially expressed in one or few tissues. SDE genes in multiple tissues are mostly linked to the sex chromosomes (Additional file 5: Table S2). (PDF 177 kb)
Additional file 5: Table S2. Genes with SDE in more than five tissues. (DOCX 17 kb)
Additional file 6: Figure S4. SDE score heatmap of 244 protein-coding X-linked genes, ordered by their chromosomal position. Three genes have men-biased expression in multiple tissues, are in the PAR1, and none in PAR2 regions (green boxes). Scores are color-coded from blue (strictly men) to red (strictly women), with non-differential expression in white. (PDF 152 kb)
Additional file 7: Figure S5. Hierarchical clustering of 44 tissues common to men and women (excluding mammary glands) by their gene SDE patterns. Percent *p*-values are Bootstrap-Probability in green, and Approximately-Unbiased in red [34]. The mammary gland tissue was excluded from the analysis because it had an order of magnitude more SDE genes than the other 44 common tissues. (PDF 45 kb)
Additional file 8
**Figure S6.** The first two components of principle component analysis of all protein-coding genes with SDE in at least one non-mammary gland tissue. Cluster colors denote groups of genes with the similar SDE patterns. See also Fig. 2. (PDF 191 kb)
Additional file 9: Figure S7. Expression of *TSHB* and *MUCL1* genes in 53 human tissues. Box-plots of women samples are in red and men samples in blue. The pituitary-specific gene *TSHB* is significantly overexpressed in men. *MUCL1* is significantly overexpressed in men skin and in women mammary glands. (PDF 199 kb)
Additional file 10
**Table S3.** Integrated SDE NOISeqBIO and NOISeq-sim based analyses (see “Methods”) for all protein-coding genes in 44 tissues common to men and women (excluding mammary glands). Values below or above zero denote men- or women-biased expression, respectively, and zero denotes genes with insignificant SDE. (CSV 2111 kb)
Additional file 11
**Figure S8.** Non-Y-linked genes partitioning around medoids clustering by the gene SDE patterns in 44 tissues common to men and women (excluding mammary glands). To identify SDE in genes with complex modes of expression we applied the NOISeq-sim approach that weighs groups of outliers (see “Methods”). The mammary gland tissue was excluded from the analysis because it had an order of magnitude more SDE genes than the other common tissues. (PDF 138 kb)
Additional file 12: Figure S9. Sex-biased expression of the *MTRNR2L2* gene. *MTRNR2L2* is notably expressed in women substantia-nigra (**a**, red box) due to overexpression in women older than 60 years. **b** Women under and above 60 years, *n* = 12 and *n* = 12 respectively. Men under and above 60 years, *n* = 16 and *n* = 23, respectively. (PDF 297 kb)
Additional file 13: Table S4. Women-biased protein-coding gene terms enrichment analyses. Multiple Excel worksheets summarizing GeneAnalytics [35] gene enrichments including diseases, GO terms, and pathways. (XLSX 625 kb)
Additional file 14: Table S5. Men-biased protein-coding gene terms enrichment analyses. Multiple Excel worksheets summarizing GeneAnalytics [35] gene enrichments including diseases, GO-terms, and pathways. (XLSX 557 kb)
Additional file 15: Table S6. SDE scores for testis overexpressed protein-coding genes across 53 tissues. Values below or above zero denote male or women-biased expression, respectively, and zero denotes genes with insignificant SDE. (CSV 791 kb)
Additional file 16: Figure S10. SDE score heatmap of testis-specific and moderately specific genes. Red and blue denote women or men specificity, respectively. (PDF 200 kb)
Additional file 17: Table S7. SDE scores for non-testis sex-specific and moderately sex-specific protein-coding genes across 53 tissues. Values below or above zero denote men- or women-biased expression, respectively. (CSV 177 kb)
Additional file 18: Figure S13. Age-related expression of the *NPPB* gene in heart left ventricle show overexpression in young women. Women under and above 60, *n* = 54 and *n* = 22, respectively. Men under and above 60, *n* = 103 and *n* = 39, respectively. (PDF 92 kb)
Additional file 19: Figure S11. Expression of *XIST* gene in 53 human tissues shown as box-plots with women samples in red and men samples in blue. Pink and light blue are women and men reproductive tissues, respectively. (PDF 41 kb)
Additional file 20: Figure S12. SDE score heatmap of non-protein-coding genes. Red and blue denote women or men specificity, respectively. (PDF 221 kb)

## References

[1] Bachtrog D, Mank JE, Peichel CL, Kirkpatrick M, Otto SP, Ashman T-L, Hahn MW, Kitano J, Mayrose I, Ming R (2014). Sex determination: why so many ways of doing it?. PLoS Biol.

[2] McClellan HL, Miller SJ, Hartmann PE (2008). Evolution of lactation: nutrition v. protection with special reference to five mammalian species. Nutr Res Rev.

[3] McClellan J, King M-C (2010). Genetic heterogeneity in human disease. Cell.

[4] Connallon T (2015). The geography of sex‐specific selection, local adaptation, and sexual dimorphism. Evolution.

[5] Deaner RO, Shepherd SV, Platt ML (2007). Familiarity accentuates gaze cuing in women but not men. Biol Lett.

[6] Goldstein JM, Holsen L, Handa R, Tobet S (2014). Fetal hormonal programming of sex differences in depression: linking women's mental health with sex differences in the brain across the lifespan. Front Neurosci.

[7] Giedd JN, Castellanos FX, Rajapakse JC, Vaituzis AC, Rapoport JL (1997). Sexual dimorphism of the developing human brain. Prog Neuro-Psychopharmacol Biol Psychiatry.

[8] Collaer ML, Hines M (1995). Human behavioral sex differences: a role for gonadal hormones during early development?. Psychol Bull.

[9] Waldron I (1983). Sex differences in human mortality: the role of genetic factors. Soc Sci Med.

[10] Subbaraman M, Goldman-Mellor S, Anderson E, LeWinn K, Saxton K, Shumway M, Catalano R (2010). An exploration of secondary sex ratios among women diagnosed with anxiety disorders. Human Reprod.

[11] Pulido MR, Rabanal-Ruiz Y, Almabouada F, Díaz-Ruiz A, Burrell MA, Vázquez MJ, Castaño JP, Kineman RD, Luque RM, Diéguez C (2013). Nutritional, hormonal, and depot-dependent regulation of the expression of the small GTPase Rab18 in rodent adipose tissue. J Mol Endocrinol.

[12] Link JC, Chen X, Arnold AP, Reue K (2013). Metabolic impact of sex chromosomes. Adipocyte.

[13] Bartley EJ, Fillingim RB (2013). Sex differences in pain: a brief review of clinical and experimental findings. Br J Anaesth.

[14] Courtright SH, McCormick BW, Postlethwaite BE, Reeves CJ, Mount MK (2013). A meta-analysis of sex differences in physical ability: revised estimates and strategies for reducing differences in selection contexts. J Appl Psychol.

[15] Tseng LA, Delmonico MJ, Visser M, Boudreau RM, Goodpaster BH, Schwartz AV, Simonsick EM, Satterfield S, Harris T, Newman AB (2014). Body composition explains sex differential in physical performance among older adults. J Gerontol Ser A Biol Med Sci.

[16] Dimas AS, Nica AC, Montgomery SB, Stranger BE, Raj T, Buil A, Giger T, Lappalainen T, Gutierrez-Arcelus M, McCarthy MI (2012). Sex-biased genetic effects on gene regulation in humans. Genome Res.

[17] Rawlik K, Canela-Xandri O, Tenesa A (2016). Evidence for sex-specific genetic architectures across a spectrum of human complex traits. Genome Biol.

[18] Gilks WP, Abbott JK, Morrow EH (2014). Sex differences in disease genetics: evidence, evolution, and detection. Trends Genet.

[19] Sandberg K, Verbalis JG (2013). Sex and the basic scientist: is it time to embrace Title IX?. Biol Sex Differ.

[20] Fisher RA (1930). The genetical theory of natural selection: a complete variorum edition.

[21] Connallon T, Clark AG (2011). The resolution of sexual antagonism by gene duplication. Genetics.

[22] Frank SA, Hurst LD (1996). Mitochondria and male disease. Nature.

[23] Morrow EH, Connallon T (2013). Implications of sex‐specific selection for the genetic basis of disease. Evol Appl.

[24] Gershoni M, Pietrokovski S (2014). Reduced selection and accumulation of deleterious mutations in genes exclusively expressed in men. Nat Commun.

[25] Innocenti P, Morrow EH (2010). The sexually antagonistic genes of Drosophila melanogaster. PLoS Biol.

[26] Su AI, Wiltshire T, Batalov S, Lapp H, Ching KA, Block D, Zhang J, Soden R, Hayakawa M, Kreiman G (2004). A gene atlas of the mouse and human protein-encoding transcriptomes. Proc Natl Acad Sci U S A.

[27] Ardlie KG, Deluca DS, Segrè AV, Sullivan TJ, Young TR, Gelfand ET, Trowbridge CA, Maller JB, Tukiainen T, Lek M (2015). The Genotype-Tissue Expression (GTEx) pilot analysis: multitissue gene regulation in humans. Science.

[28] Melé M, Ferreira PG, Reverter F, DeLuca DS, Monlong J, Sammeth M, Young TR, Goldmann JM, Pervouchine DD, Sullivan TJ (2015). The human transcriptome across tissues and individuals. Science.

[29] Mank JE. The transcriptional architecture of phenotypic dimorphism. Nature Ecology & Evolution. 2017;1:0006. 10.1038/s41559-016-0006 28812569

[30] Carithers LJ, Ardlie K, Barcus M, Branton PA, Britton A, Buia SA, Compton CC, DeLuca DS, Peter-Demchok J, Gelfand ET (2015). A novel approach to high-quality postmortem tissue procurement: The GTEx Project. Biopreservation Biobanking.

[31] Tarazona S, Furió-Tarí P, Turrà D, Di Pietro A, Nueda MJ, Ferrer A, Conesa A (2015). Data quality aware analysis of differential expression in RNA-seq with NOISeq R/Bioc package. Nucleic Acids Res.

[32] Tarazona S, García-Alcalde F, Dopazo J, Ferrer A, Conesa A (2011). Differential expression in RNA-seq: a matter of depth. Genome Res.

[33] Mangs HA, Morris BJ (2007). The human pseudoautosomal region (PAR): origin, function and future. Curr Genomics.

[34] Suzuki R, Shimodaira H (2006). Pvclust: an R package for assessing the uncertainty in hierarchical clustering. Bioinformatics.

[35] Fuchs SB-A, Lieder I, Stelzer G, Mazor Y, Buzhor E, Kaplan S, Bogoch Y, Plaschkes I, Shitrit A, Rappaport N (2016). GeneAnalytics: an integrative gene set analysis tool for next generation sequencing, RNAseq and microarray data. OMICS.

[36] Holditch SJ, Schreiber CA, Burnett JC, Ikeda Y (2016). Arterial remodeling in B-type natriuretic peptide knock-out females. Sci Rep.

[37] Wang TJ, Larson MG, Levy D, Leip EP, Benjamin EJ, Wilson PW, Sutherland P, Omland T, Vasan RS (2002). Impact of age and sex on plasma natriuretic peptide levels in healthy adults. Am J Cardiol.

[38] Clark BC, Collier SR, Manini TM, Ploutz-Snyder LL (2005). Sex differences in muscle fatigability and activation patterns of the human quadriceps femoris. Eur J Appl Physiol.

[39] Russ DW, Kent-Braun JA (2003). Sex differences in human skeletal muscle fatigue are eliminated under ischemic conditions. J Appl Physiol.

[40] Ellegren H, Parsch J (2007). The evolution of sex-biased genes and sex-biased gene expression. Nat Rev Genet.

[41] Abecasis GR, Altshuler D, Auton A, Brooks LD, Durbin RM, Gibbs RA, Hurles ME, McVean GA, 1000 Genomes Project Consortium (2010). A map of human genome variation from population-scale sequencing. Nature.

[42] Kryazhimskiy S, Plotkin JB (2008). The population genetics of dN/dS. PLoS Genet.

[43] Ostrow SL, Barshir R, DeGregori J, Yeger-Lotem E, Hershberg R (2014). Cancer evolution is associated with pervasive positive selection on globally expressed genes. PLoS Genet.

[44] Tennessen JA, Bigham AW, O’Connor TD, Fu W, Kenny EE, Gravel S, McGee S, Do R, Liu X, Jun G (2012). Evolution and functional impact of rare coding variation from deep sequencing of human exomes. Science.

[45] Wu R, Lin M (2006). Functional mapping - how to map and study the genetic architecture of dynamic complex traits. Nat Rev Genet.

[46] de Moura Souza A, Sichieri R (2011). Association between serum TSH concentration within the normal range and adiposity: a review. Eur J Endocrinol.

[47] Skorupskaite K, George JT, Anderson RA (2014). The kisspeptin-GnRH pathway in human reproductive health and disease. Hum Reprod Update.

[48] Guengerich FP, Waterman MR, Egli M (2016). Recent structural insights into cytochrome P450 function. Trends Pharmacol Sci.

[49] Lamba V, Lamba J, Yasuda K, Strom S, Davila J, Hancock ML, Fackenthal JD, Rogan PK, Ring B, Wrighton SA (2003). Hepatic CYP2B6 expression: gender and ethnic differences and relationship to CYP2B6 genotype and CAR (constitutive androstane receptor) expression. J Pharmacol Exp Ther.

[50] Xiong Q, Jiao Y, Hasty KA, Canale ST, Stuart JM, Beamer WG, Deng H-W, Baylink D, Gu W (2009). Quantitative trait loci, genes, and polymorphisms that regulate bone mineral density in mouse. Genomics.

[51] Brain S, Williams T, Tippins J, Morris H, MacIntyre I (1985). Calcitonin gene-related peptide is a potent vasodilator. Nature.

[52] Gangula PR, Zhao H, Supowit SC, Wimalawansa SJ, Dipette DJ, Westlund KN, Gagel RF, Yallampalli C (2000). Increased blood pressure in α-calcitonin gene–related peptide/calcitonin gene knockout mice. Hypertension.

[53] Bateman AJ (1948). Intra-sexual selection in Drosophila. Heredity.

[54] Cerase A, Pintacuda G, Tattermusch A, Avner P (2015). Xist localization and function: new insights from multiple levels. Genome Biol.

[55] Kassam I, Lloyd-Jones L, Holloway A, Small KS, Zeng B, Bakshi A, Metspalu A, Gibson G, Spector TD, Esko T (2016). Autosomal genetic control of human gene expression does not differ across the sexes. Genome Biol.

[56] Chen C-Y, Lopes-Ramos CM, Kuijjer ML, Paulson JN, Sonawane AR, Fagny M, Platig J, Glass K, Quackenbush J, DeMeo DL. Sexual dimorphism in gene expression and regulatory networks across human tissues. bioRxiv 2016. Epub ahead of print. doi:10.1101/082289. 10.1016/j.celrep.2020.107795 PMC7898458 32579922

[57] Herrero J, Muffato M, Beal K, Fitzgerald S, Gordon L, Pignatelli M, Vilella AJ, Searle SM, Amode R, Brent S (2016). Ensembl comparative genomics resources. Database.

[58] Wang K, Li M, Hakonarson H (2010). ANNOVAR: functional annotation of genetic variants from high-throughput sequencing data. Nucleic Acids Res.

[59] Kumar P, Henikoff S, Ng PC (2009). Predicting the effects of coding non-synonymous variants on protein function using the SIFT algorithm. Nat Protoc.

[60] Adzhubei IA, Schmidt S, Peshkin L, Ramensky VE, Gerasimova A, Bork P, Kondrashov AS, Sunyaev SR (2010). A method and server for predicting damaging missense mutations. Nat Methods.

[61] Lek M, Karczewski KJ, Minikel EV, Samocha KE, Banks E, Fennell T, O’Donnell-Luria AH, Ware JS, Hill AJ, Cummings BB (2016). Analysis of protein-coding genetic variation in 60,706 humans. Nature.

[62] Efron B, Tibshirani R, Storey JD, Tusher V (2001). Empirical Bayes analysis of a microarray experiment. J Am Stat Assoc.

[63] Ferin M, Jewelewicz R (1993). The menstrual cycle: physiology, reproductive disorders, and infertility.

[64] Love MI, Huber W, Anders S (2014). Moderated estimation of fold change and dispersion for RNA-seq data with DESeq2. Genome Biol.

[65] Kaufman L, Rousseeuw PJ. Finding groups in data: an introduction to cluster analysis, vol. 344. Hoboken, New Jersey: John Wiley & Sons; 2009.

