# Supplementary figures and images for: The landscape of sex-differential transcriptome and its consequent selection in human adults
Source: BMC Biol. 2017 Feb 7;15:7. doi: 10.1186/s12915-017-0352-z (PMC5297171; doi:10.1186/s12915-017-0352-z)

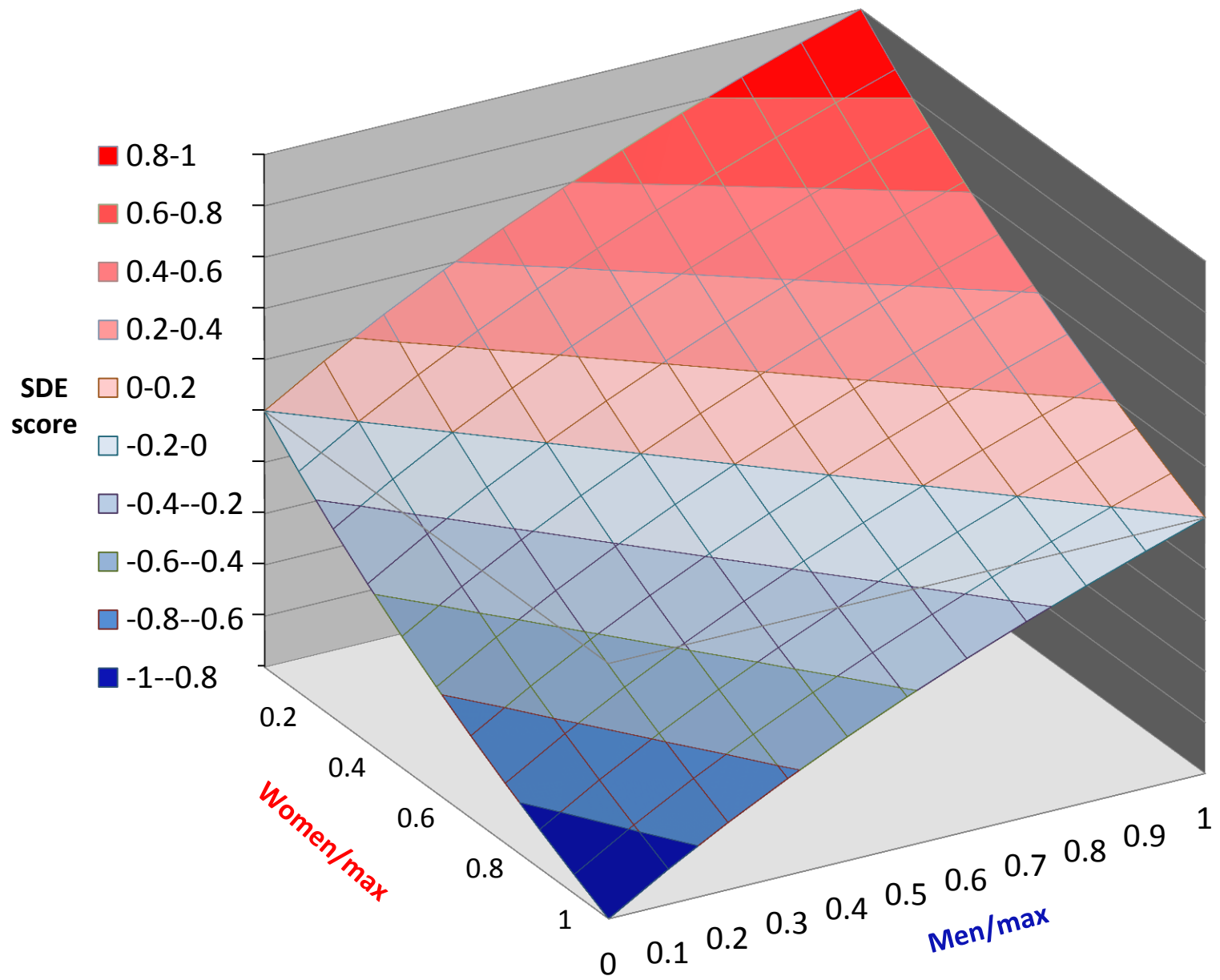

Supplement: Additional file 1: Figure S1. — Differential expression score. Landscape of scores for all possible ratios of men and women expression values using a base 2 logarithm. The formula we derived for SDE score can be generalized to compare the difference between two populations (x and y) in a certain tissue or condition (t), normalizing by a gene (g) maximal expression value (MAXg). This differential expression score (DES) is a logarithm of the normalized ratios, giving scores between −1 and 1. We use a logarithm base of 2, but other bases (n) are possible. The general expression is thus DES = LOGn{(1 + (EXPRg, t x/MAXg) * (n − 1))/(1 + (EXPRg, t y/MAXg) * (n − 1))} where EXPRg, t x is the expression value of gene g in tissue/condition t for population x. This score returns the differential expression value of a gene in specific tissue/condition, relative to the maximal expression of the gene. The value ranges from 1 (exclusive expression in x) to −1 (exclusive expression in y). Larger logarithm bases (n) exponentially increase the transitions between exclusive expression (1 and −1) and non-differential (0) scores. (PDF 276 kb) [file 12915_2017_352_MOESM1_ESM.pdf]

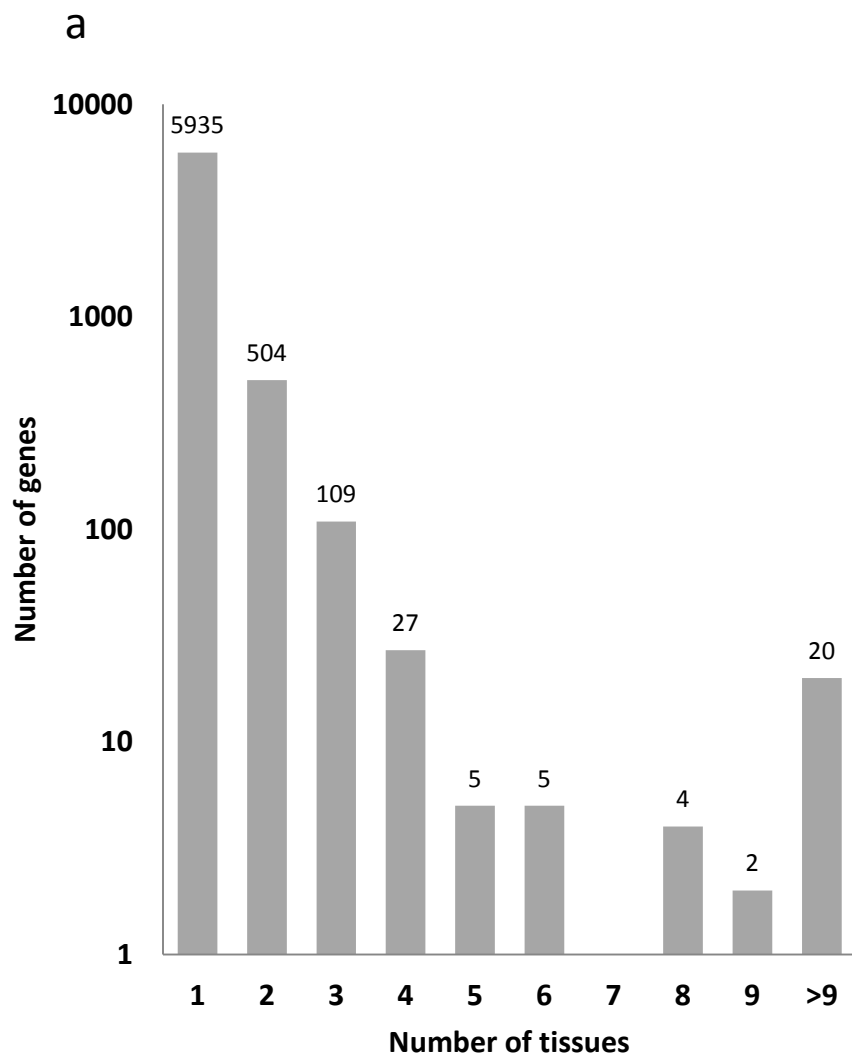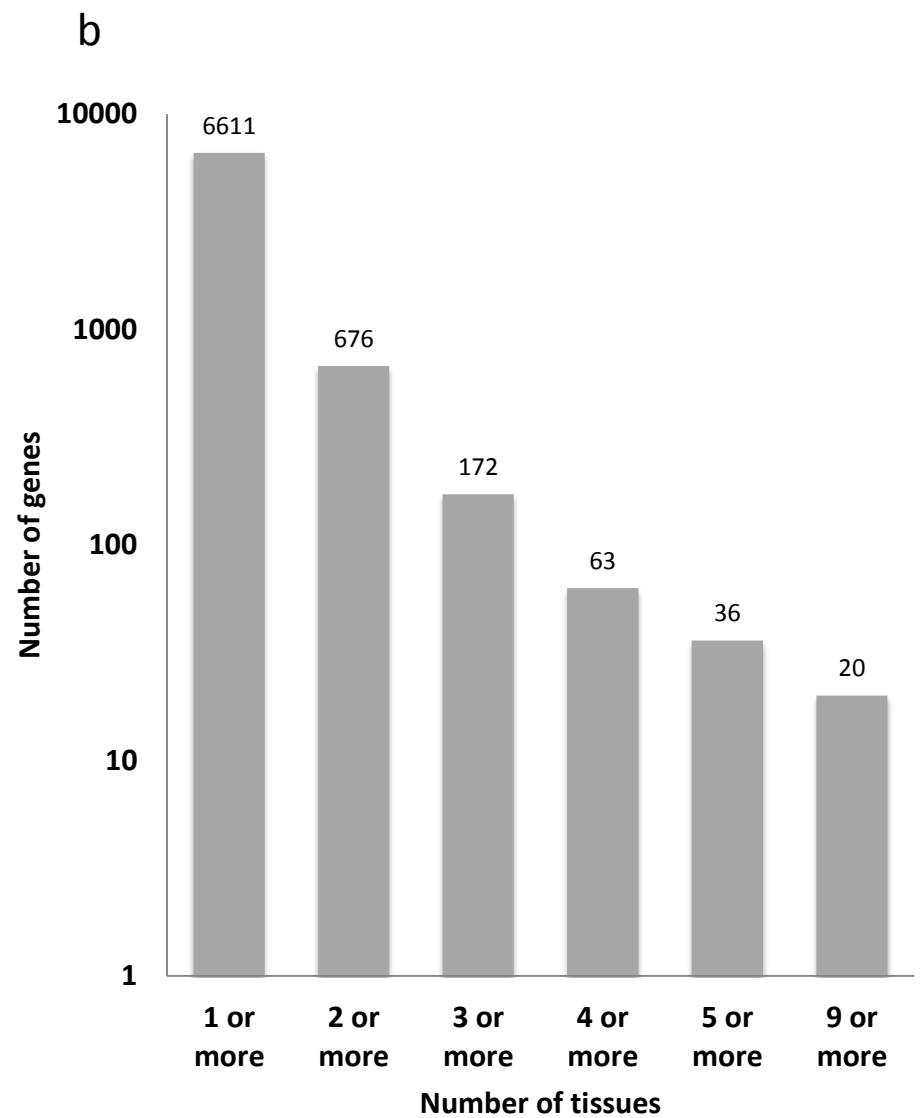

Supplement: Additional file 4: Figure S3. — Occurrence of genes according to number and exact (a) or cumulative (b) number of tissues they have SDE in. Most SDE genes are differentially expressed in one or few tissues. SDE genes in multiple tissues are mostly linked to the sex chromosomes (Additional file 5: Table S2). (PDF 177 kb) [file 12915_2017_352_MOESM4_ESM.pdf]

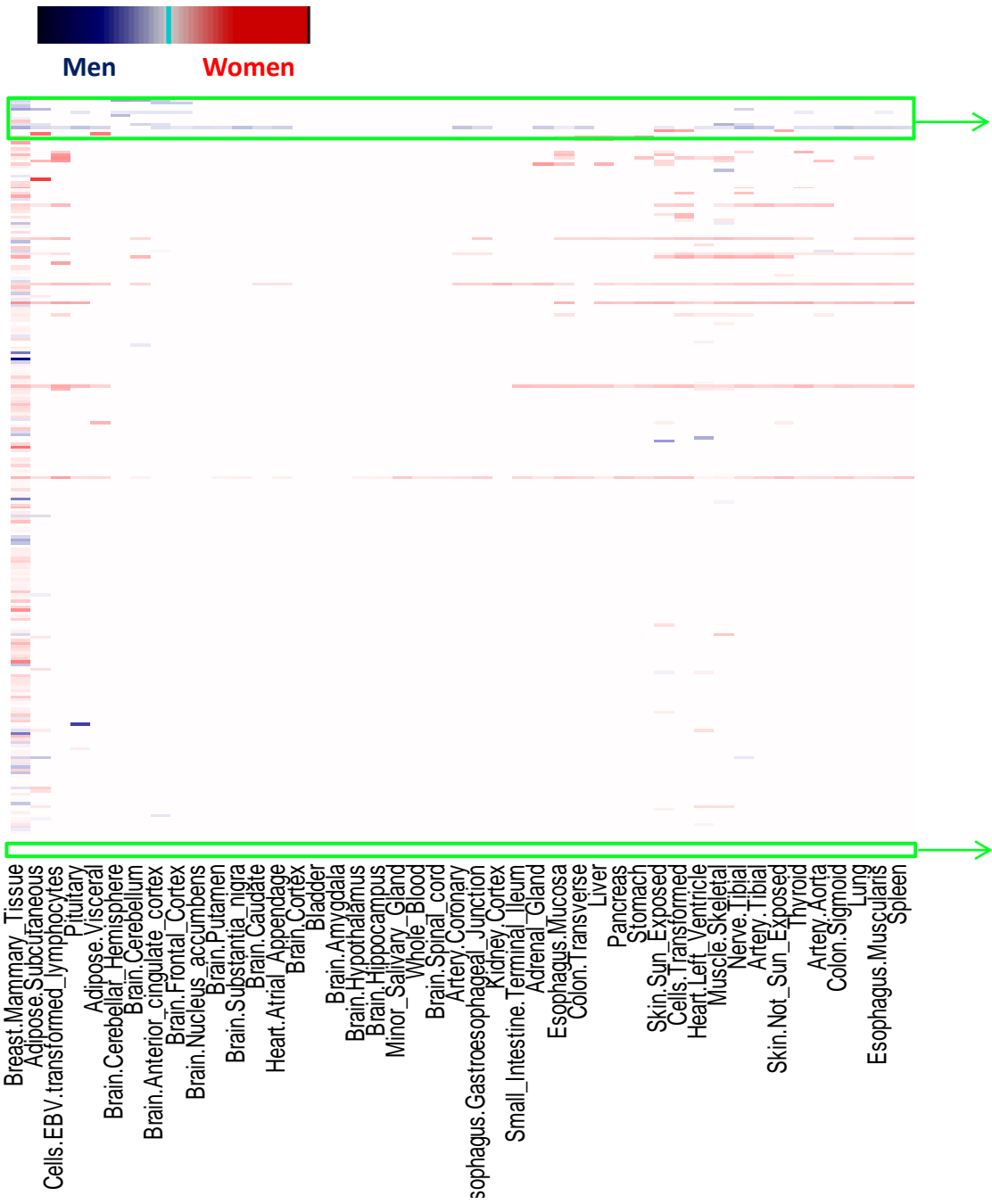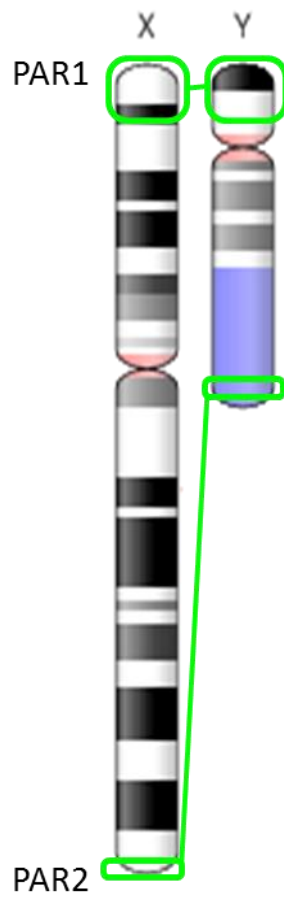

Supplement: Additional file 6: Figure S4. — SDE score heatmap of 244 protein-coding X-linked genes, ordered by their chromosomal position. Three genes have men-biased expression in multiple tissues, are in the PAR1, and none in PAR2 regions (green boxes). Scores are color-coded from blue (strictly men) to red (strictly women), with non-differential expression in white. (PDF 152 kb) [file 12915_2017_352_MOESM6_ESM.pdf]

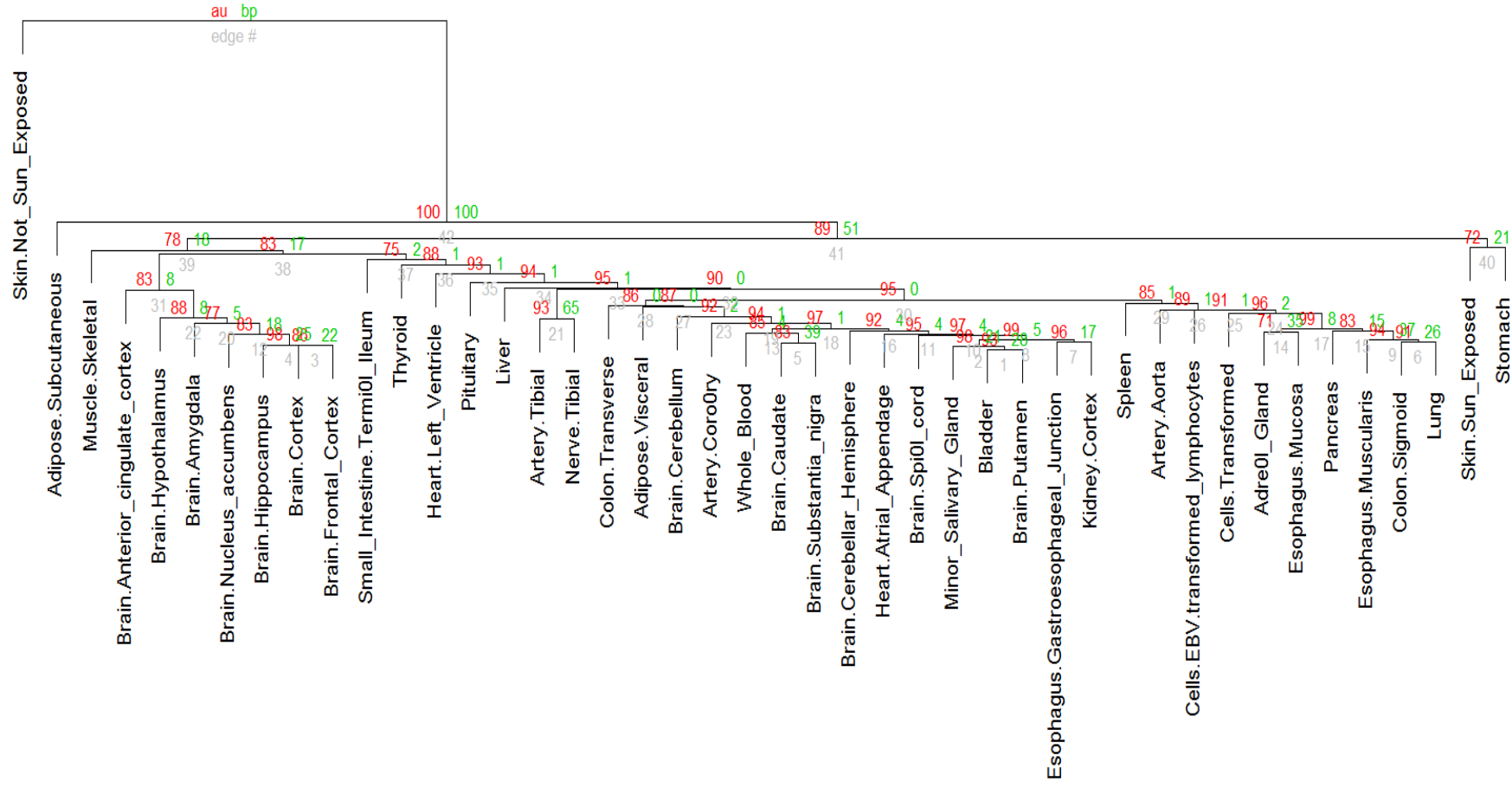

Supplement: Additional file 7: Figure S5. — Hierarchical clustering of 44 tissues common to men and women (excluding mammary glands) by their gene SDE patterns. Percent p-values are Bootstrap-Probability in green, and Approximately-Unbiased in red [34]. The mammary gland tissue was excluded from the analysis because it had an order of magnitude more SDE genes than the other 44 common tissues. (PDF 45 kb) [file 12915_2017_352_MOESM7_ESM.pdf]

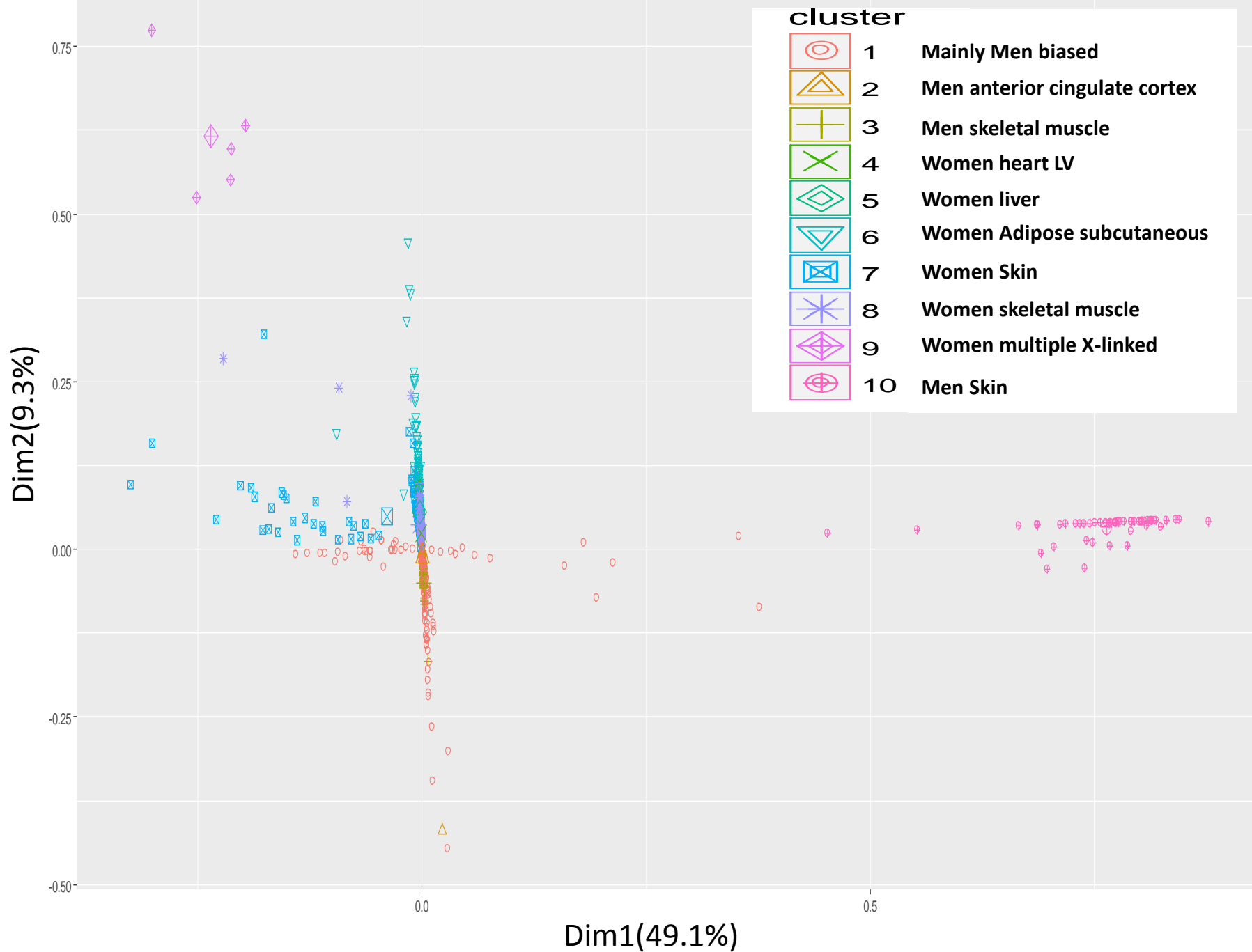

Supplement: Additional file 8 — Figure S6. The first two components of principle component analysis of all protein-coding genes with SDE in at least one non-mammary gland tissue. Cluster colors denote groups of genes with the similar SDE patterns. See also Fig. 2. (PDF 191 kb) [file 12915_2017_352_MOESM8_ESM.pdf]

RPKM

TSHB

MUCL1

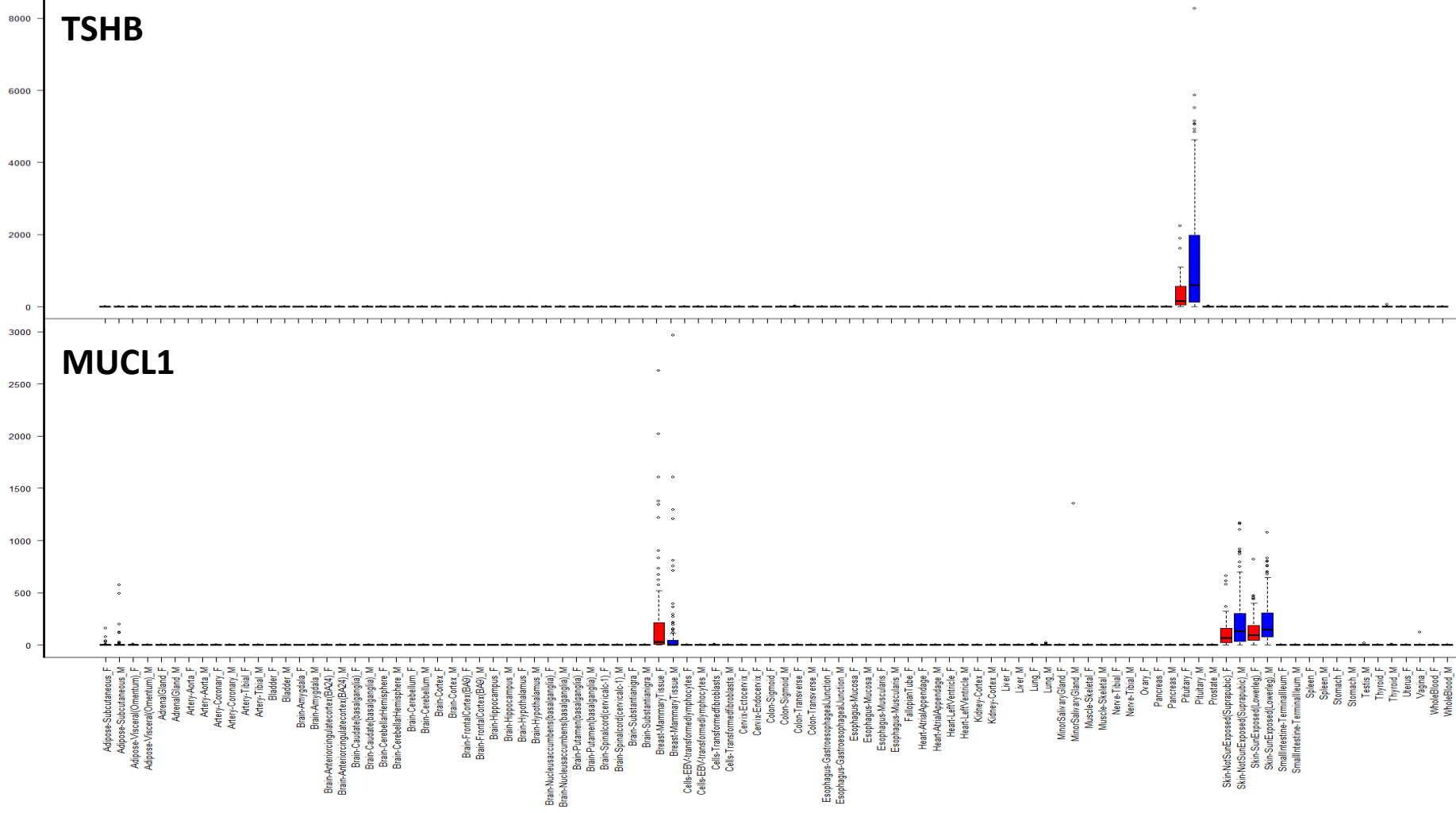

Supplement: Additional file 9: Figure S7. — Expression of TSHB and MUCL1 genes in 53 human tissues. Box-plots of women samples are in red and men samples in blue. The pituitary-specific gene TSHB is significantly overexpressed in men. MUCL1 is significantly overexpressed in men skin and in women mammary glands. (PDF 199 kb) [file 12915_2017_352_MOESM9_ESM.pdf]

Dim2(6.8%)

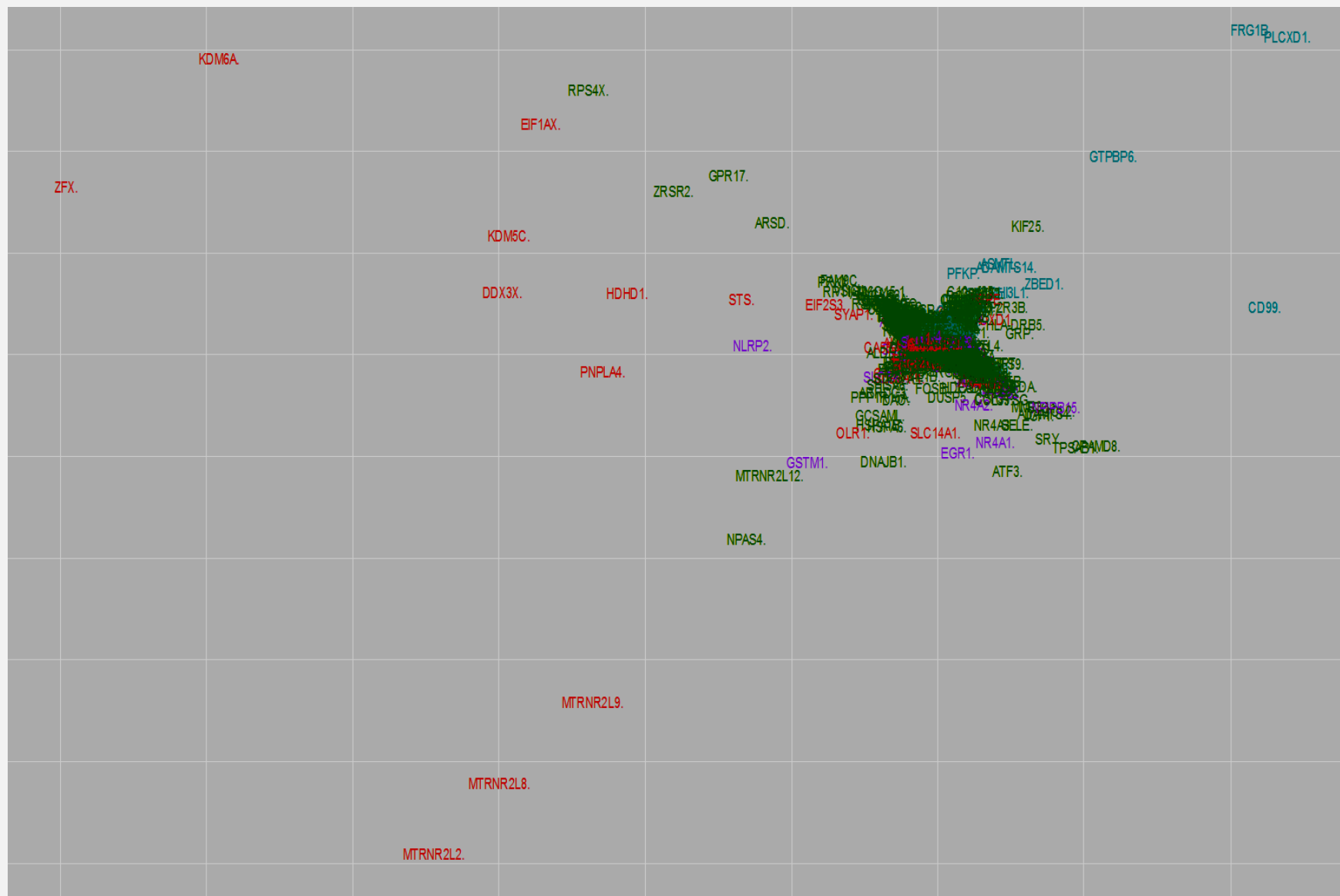

Dim1(12.1%)

Supplement: Additional file 11 — Figure S8. Non-Y-linked genes partitioning around medoids clustering by the gene SDE patterns in 44 tissues common to men and women (excluding mammary glands). To identify SDE in genes with complex modes of expression we applied the NOISeq-sim approach that weighs groups of outliers (see “Methods”). The mammary gland tissue was excluded from the analysis because it had an order of magnitude more SDE genes than the other common tissues. (PDF 138 kb) [file 12915_2017_352_MOESM11_ESM.pdf]

**a**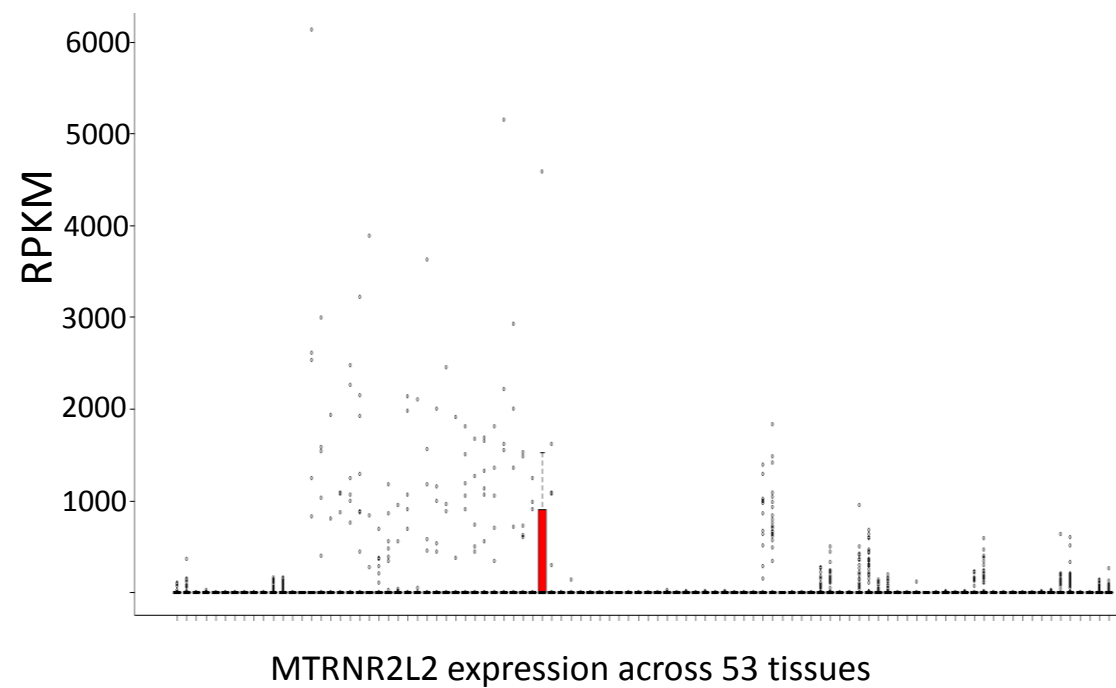**b**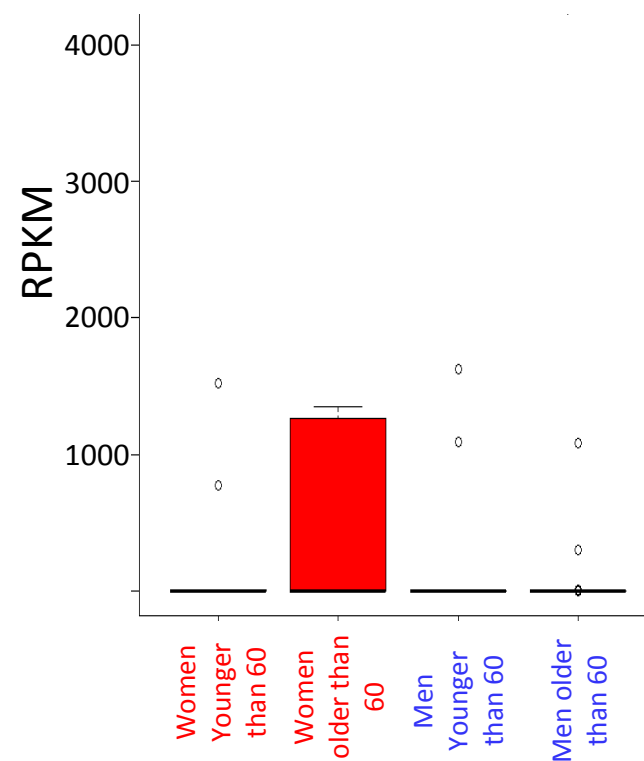

Supplement: Additional file 12: Figure S9. — Sex-biased expression of the MTRNR2L2 gene. MTRNR2L2 is notably expressed in women substantia-nigra (a, red box) due to overexpression in women older than 60 years. b Women under and above 60 years, n = 12 and n = 12 respectively. Men under and above 60 years, n = 16 and n = 23, respectively. (PDF 297 kb) [file 12915_2017_352_MOESM12_ESM.pdf]

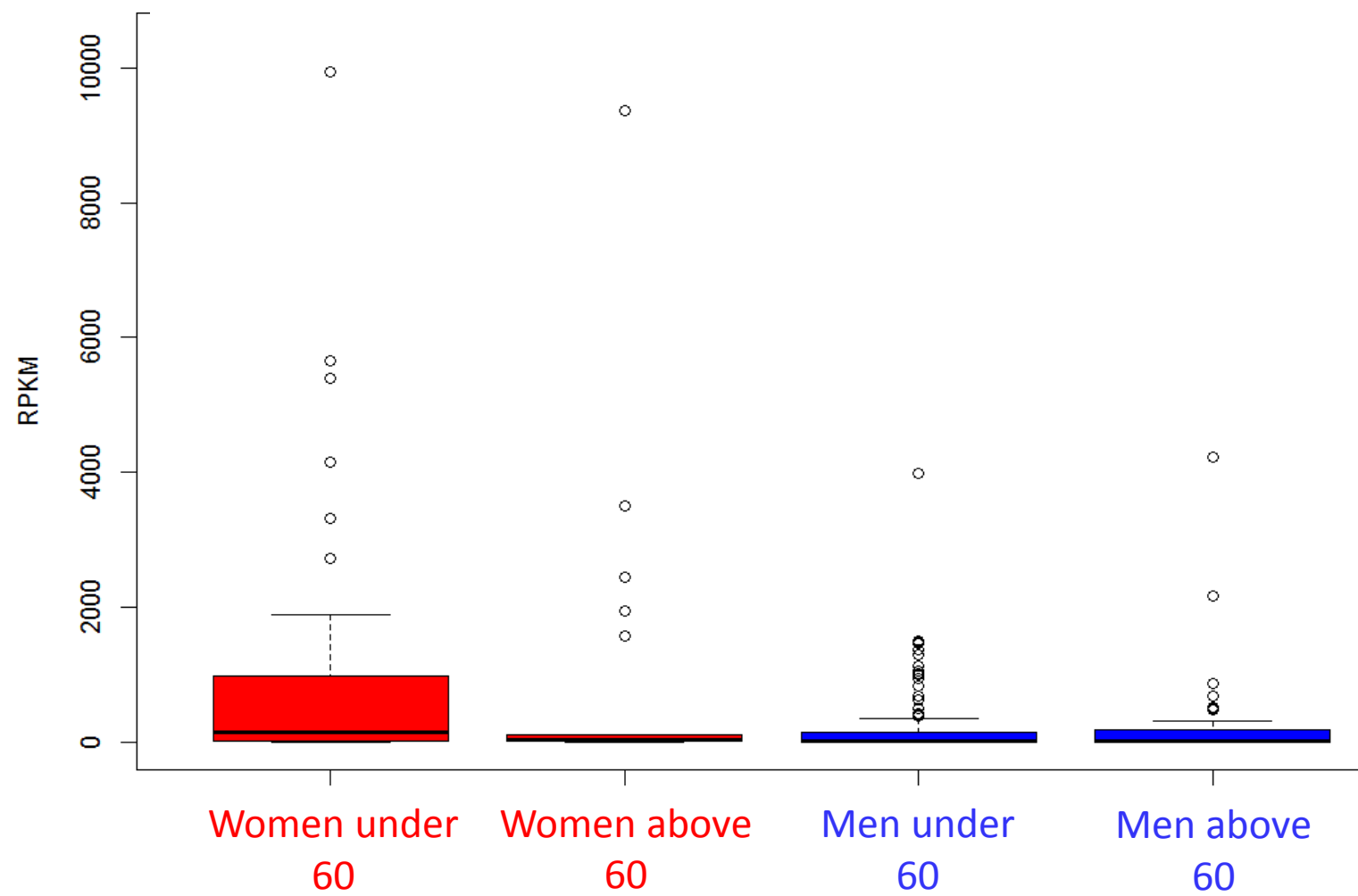

Supplement: Additional file 18: Figure S13. — Age-related expression of the NPPB gene in heart left ventricle show overexpression in young women. Women under and above 60, n = 54 and n = 22, respectively. Men under and above 60, n = 103 and n = 39, respectively. (PDF 92 kb) [file 12915_2017_352_MOESM18_ESM.pdf]

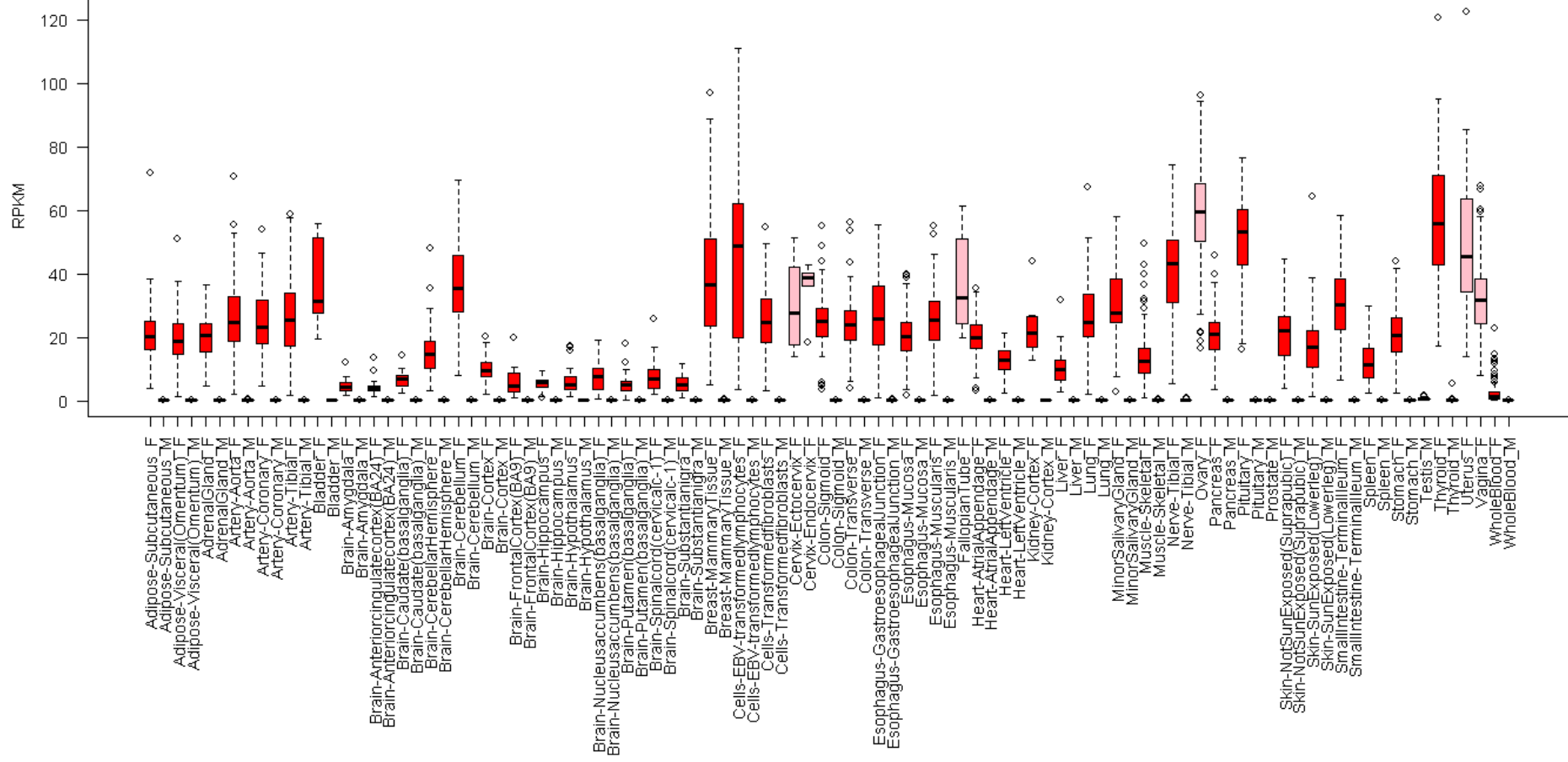

Supplement: Additional file 19: Figure S11. — Expression of XIST gene in 53 human tissues shown as box-plots with women samples in red and men samples in blue. Pink and light blue are women and men reproductive tissues, respectively. (PDF 41 kb) [file 12915_2017_352_MOESM19_ESM.pdf]

# Genes

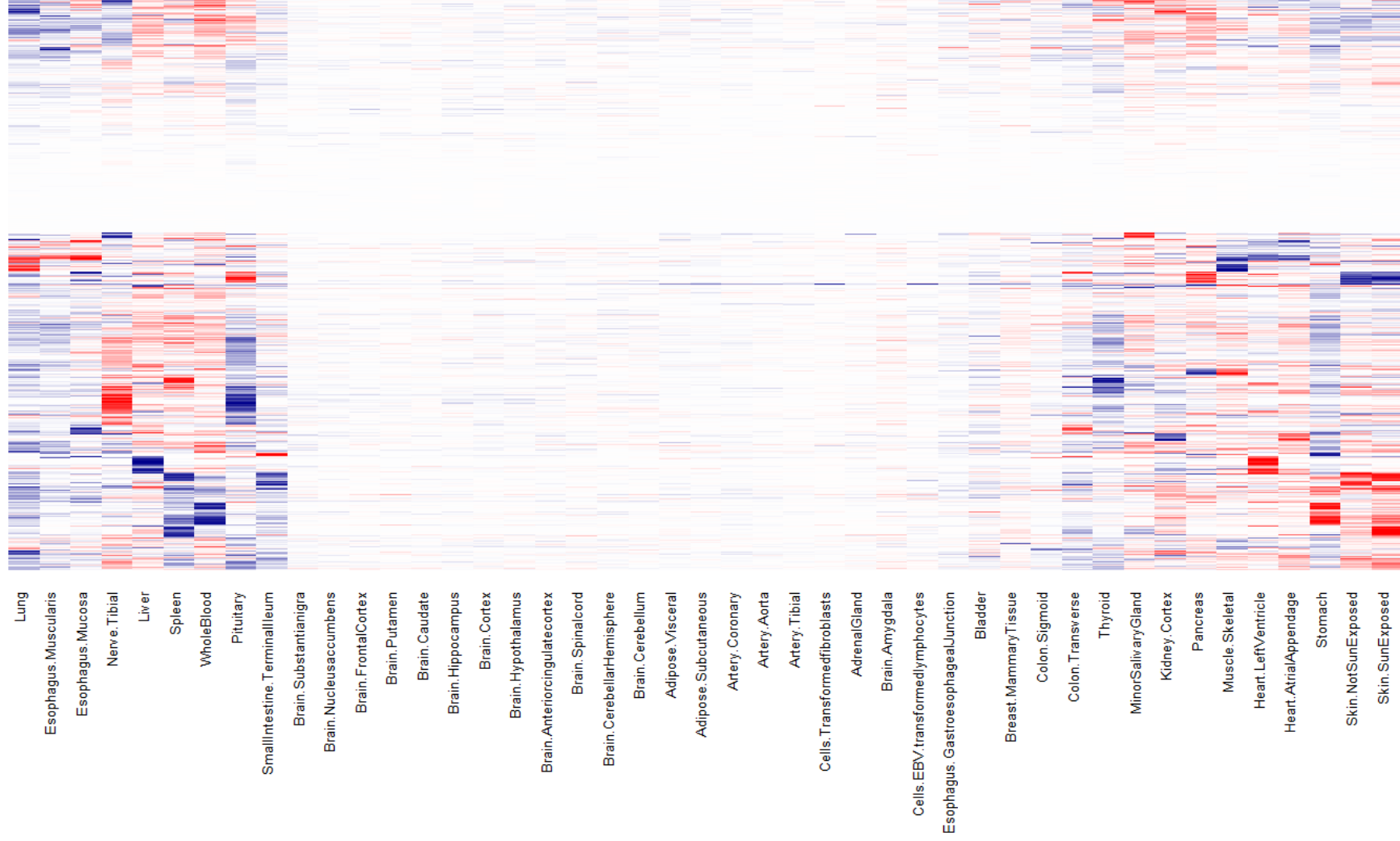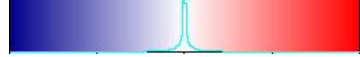

Male

Female

Supplement: Additional file 20: Figure S12. — SDE score heatmap of non-protein-coding genes. Red and blue denote women or men specificity, respectively. (PDF 221 kb) [file 12915_2017_352_MOESM20_ESM.pdf]
