# Supplementary material for: The landscape of sex-differential transcriptome and its consequent selection in human adults
Source: BMC Biol. 2017 Feb 7;15:7. doi: 10.1186/s12915-017-0352-z (PMC5297171; doi:10.1186/s12915-017-0352-z)
Supplement: Additional file 2: Figure S2. — SDE score heatmap of all protein-coding genes in 45 tissues common to both sexes. Scores are color-coded from blue (strictly men) to red (strictly women), with non-differential expression in white. Most genes are similarly expressed in most tissues with the exception of the breast mammary gland (more than 6000 SDE genes). (PDF 187 kb) [file 12915_2017_352_MOESM2_ESM.pdf]

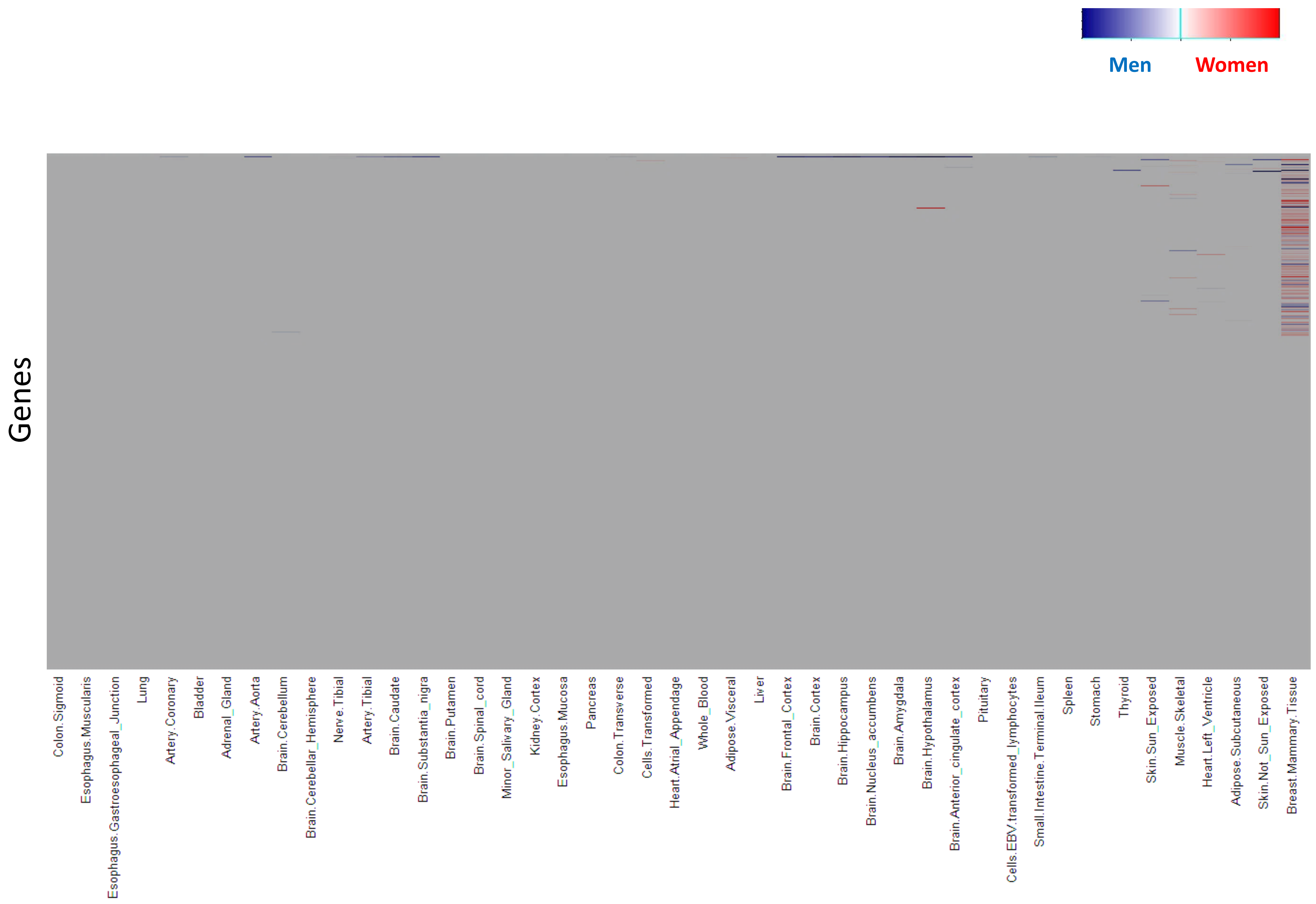

Men
Women
Genes
Colon.Sigmoid
Esophagus.Muscularis
Esophagus.Gastroesophageal_Junction
Lung
Artery.Coronary
Bladder
Adrenal_Gland
Artery.Aorta
Brain.Cerebellum
Brain.Cerebellar_Hemisphere
Nerve.Tibial
Artery.Tibial
Brain.Caudate
Brain.Substantia_nigra
Brain.Putamen
Brain.Spinal_cord
Minor_Salivary_Gland
Kidney.Cortex
Esophagus.Mucosa
Pancreas
Colon.Transverse
Cells.Transformed
Heart.Atrial_Appendage
Whole_Blood
Adipose.Visceral
Liver
Brain.Frontal_Cortex
Brain.Cortex
Brain.Hippocampus
Brain.Nucleus_accumbens
Brain.Amygdala
Brain.Hypothalamus
Brain.Anterior_cingulate_cortex
Pituitary
Cells.EBV.transformed_lymphocytes
Small.Intestine.Terminal.Ileum
Spleen
Stomach
Thyroid
Skin.Sun_Exposed
Muscle.Skeletal
Heart.Left_Ventricle
Adipose.Subcutaneous
Skin.Not_Sun_Exposed
Breast.Mammary.Tissue
