# Supplementary material for: The landscape of sex-differential transcriptome and its consequent selection in human adults
Source: BMC Biol. 2017 Feb 7;15:7. doi: 10.1186/s12915-017-0352-z (PMC5297171; doi:10.1186/s12915-017-0352-z)
Supplement: Additional file 5: Table S2. — Genes with SDE in more than five tissues. (DOCX 17 kb) [file 12915_2017_352_MOESM5_ESM.docx]

Table S2: Genes with SDE in more than 5 tissues.

| **Gene** | **Chromosome** | **Tissues** |
| --- | --- | --- |
| DDX3Y | Y | 45 |
| EIF1AY | Y | 45 |
| KDM5D | Y | 45 |
| RPS4Y1 | Y | 45 |
| TMSB4Y | Y | 45 |
| USP9Y | Y | 45 |
| UTY | Y | 45 |
| ZFY | Y | 45 |
| NLGN4Y | Y | 44 |
| RPS4X | X | 36 |
| DDX3X | X | 30 |
| TBL1Y | Y | 29 |
| CD99 | X PAR1* | 28 |
| KDM5C | X | 25 |
| EIF1AX | X | 21 |
| KDM6A | X | 21 |
| SRY | Y | 19 |
| EIF2S3 | X | 18 |
| PCDH11Y | Y | 17 |
| ZRSR2 | X | 11 |
| CES1 | 16 | 9 |
| ZFX | X | 9 |
| HDHD1 | X | 8 |
| MMP3 | 11 | 8 |
| SLC25A6 | X PAR1* | 8 |
| UBA1 | X | 8 |
| ARSD | X | 6 |
| LTBP4 | 19 | 6 |
| MAP7D2 | X | 6 |
| PRKX | X | 6 |
| ZBED1 | X PAR1* | 6 |

* PAR1 genes with identical sequences in their X and Y copies
