# Supplementary material for: The landscape of sex-differential transcriptome and its consequent selection in human adults
Source: BMC Biol. 2017 Feb 7;15:7. doi: 10.1186/s12915-017-0352-z (PMC5297171; doi:10.1186/s12915-017-0352-z)
Supplement: Additional file 16: Figure S10. — SDE score heatmap of testis-specific and moderately specific genes. Red and blue denote women or men specificity, respectively. (PDF 200 kb) [file 12915_2017_352_MOESM16_ESM.pdf]

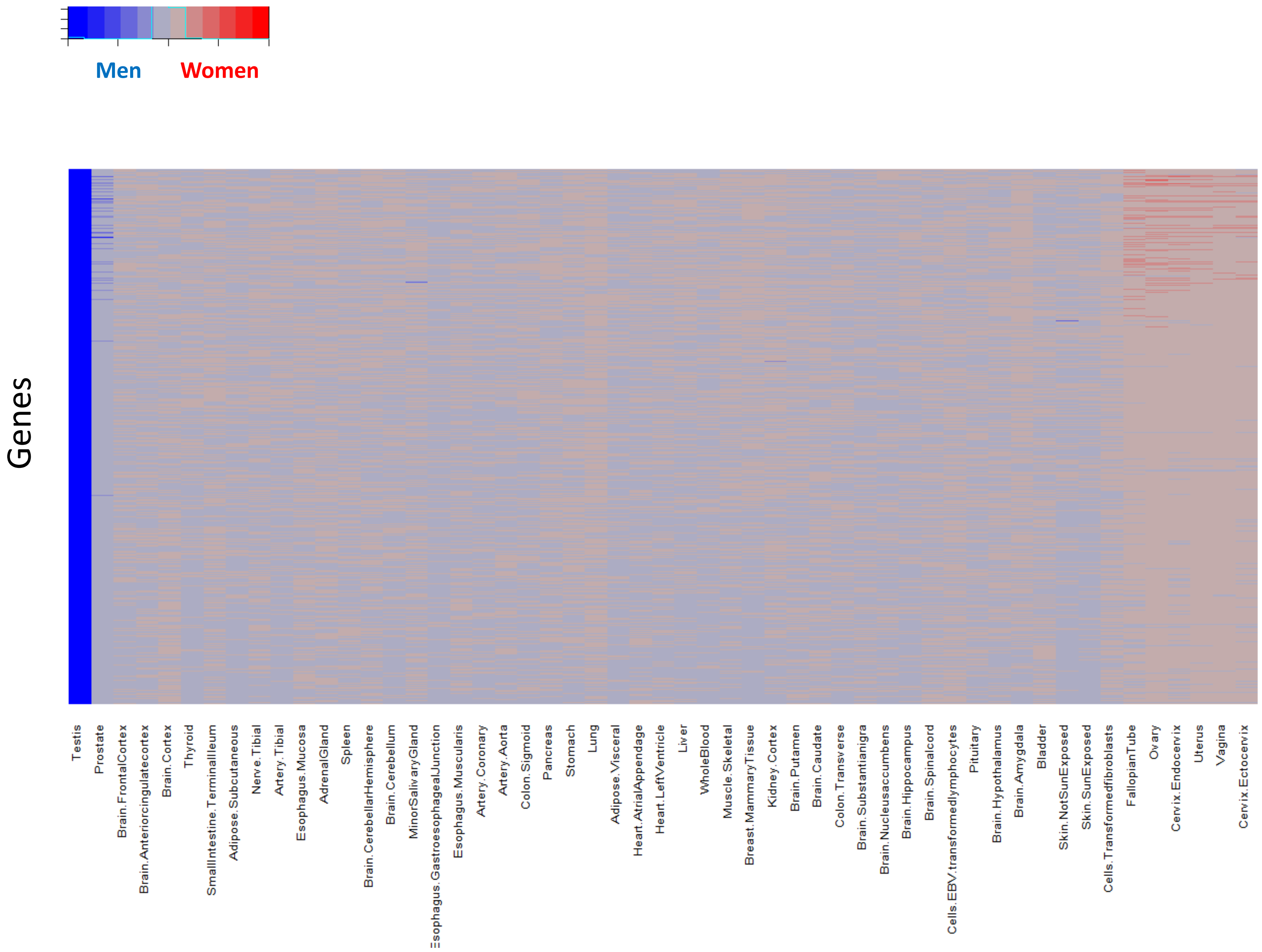

Men
Women
Genes
Testis
Prostate
Brain.FrontalCortex
Brain.Anteriorcingulatecortex
Brain.Cortex
Thyroid
SmallIntestine.TerminalIleum
Adipose.Subcutaneous
Nerve.Tibial
Artery.Tibial
Esophagus.Mucosa
AdrenalGland
Spleen
Brain.CerebellarHemisphere
Brain.Cerebellum
MinorSalivaryGland
Esophagus.GastroesophagealJunction
Esophagus.Muscularis
Artery.Coronary
Artery.Aorta
Colon.Sigmoid
Pancreas
Stomach
Lung
Adipose.Visceral
Heart.AtrialAppendage
Heart.LeftVentricle
Liver
WholeBlood
Muscle.Skeletal
Breast.MammaryTissue
Kidney.Cortex
Brain.Putamen
Brain.Caudate
Colon.Transverse
Brain.Substantianigra
Brain.Nucleusaccumbens
Brain.Hippocampus
Brain.Spinalcord
Cells.EBV.transformedlymphocytes
Pituitary
Brain.Hypothalamus
Brain.Amygdala
Bladder
Skin.NotSunExposed
Skin.SunExposed
Cells.Transformedfibroblasts
FallopianTube
Ovary
Cervix.Endocervix
Uterus
Vagina
Cervix.Ectocervix
